# Supplementary material for: Proton Control of Transitions in an Amino Acid Transporter
Source: Biophys J. 2019 Aug 26;117(7):1342–51. doi: 10.1016/j.bpj.2019.07.056 (PMC6818167; doi:10.1016/j.bpj.2019.07.056)
Supplement: Document S1. Figs. S1–S10 [file mmc1.pdf]

Biophysical Journal, Volume *117*

Supplemental Information

Proton Control of Transitions in an Amino Acid Transporter

Zhiyi Wu, Irfan Alibay, Simon Newstead, and Philip C. Biggin

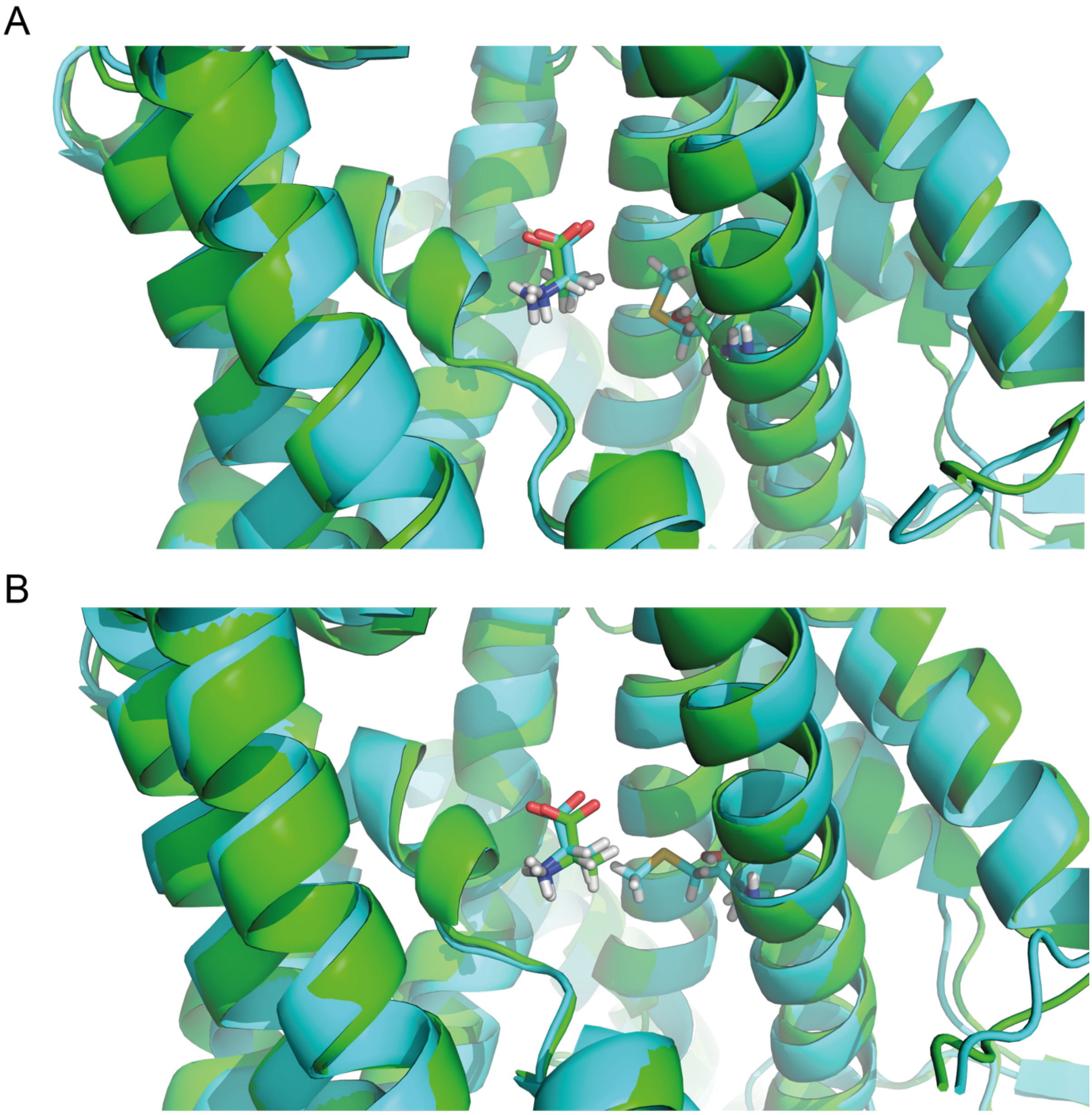

Figure S1. Comparison of M321S mutant structures to wildtype. (**A**) M321S mutant structure (green) overlaid onto wildtype (blue) inward-occluded structure (RMSD = 1.27 Å). (**B**) Comparison of M321S mutant (green) to wildtype (blue) for the inward-open conformation (RMSD = 1.10 Å).

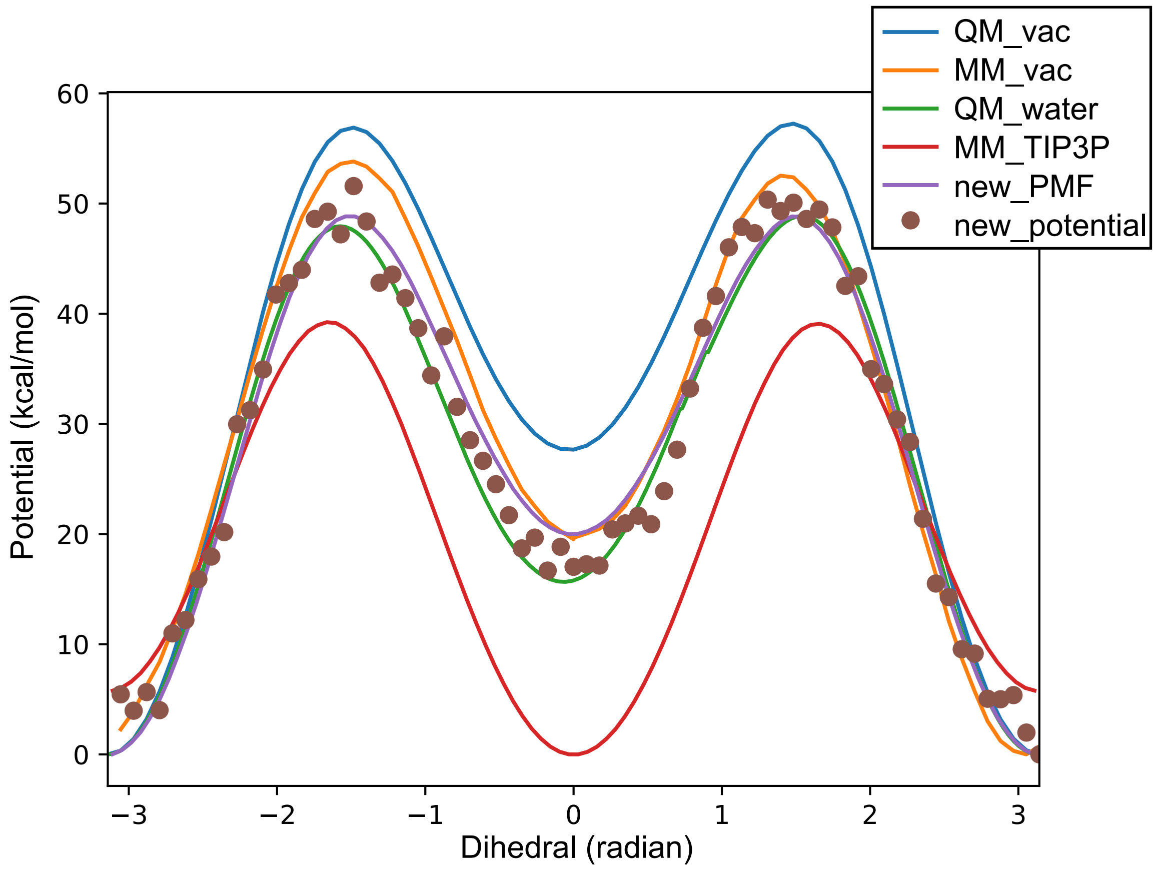

Figure S2. Potential energy surface of the C-C-O-H dihedral for a protonated glutamate residue (atoms CG-CD-OE2-HE2). Though the potential energy surface of the C-C-O-H dihedral in the Amber99SB-ILDN force field (orange line) is relatively consistent with the QM energy surface calculated at the HF/6-31G(d) level (blue line) in vacuum, the minimum dihedral angle is flipped by 180 degrees when solvated with TIP3P explicit water (red line), which is different from the QM energy surface calculated at the same level of theory with implicit PCM water (green line). The refitted dihedral reproduces the QM energy surface both in terms of the total system potential (brown dot) or the PMF profile (purple line) when solvated with TIP3P water. The new dihedral potential is represented using the Ryckaert-Bellemans dihedral, which has the functional form

$$\frac{1}{2}[F_1(1+\cos\varphi)+F_2(1-\cos 2\varphi)+F_3(1+\cos 3\varphi)]$$

where F_1 = 34.8164509, F_2 = -13.0191847 and F_3 = -21.7972662 (kJ mol^{-1}).

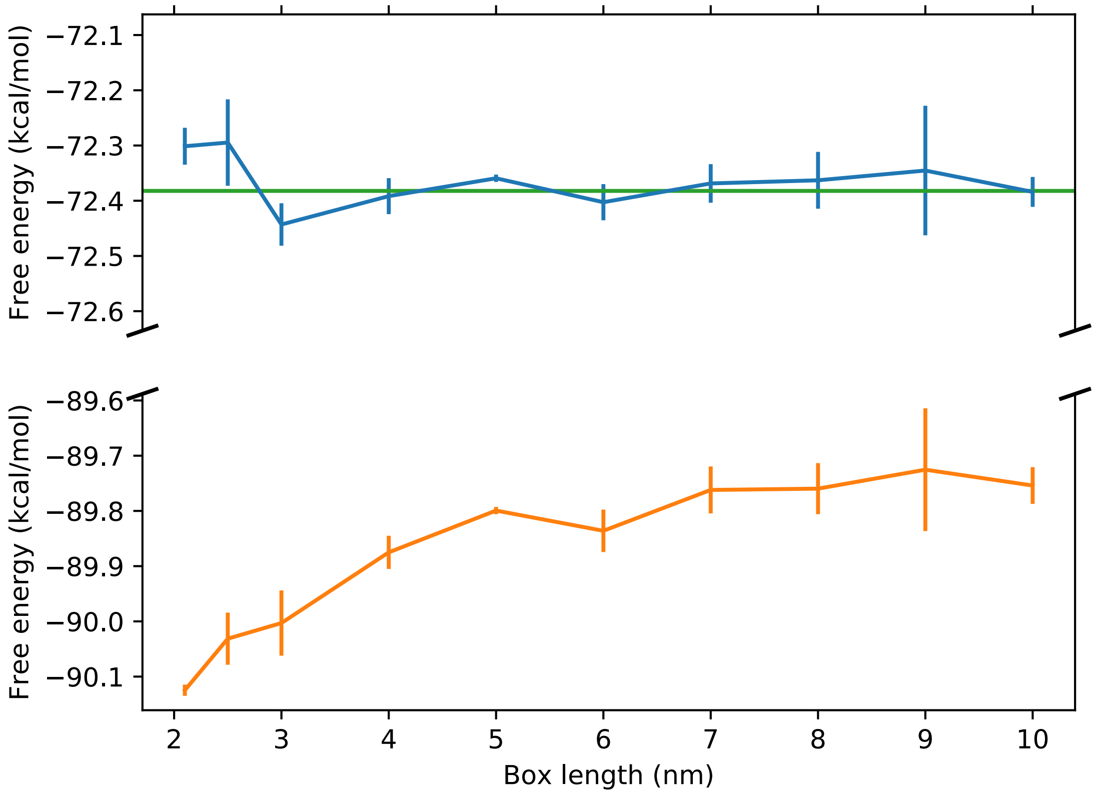

Figure S3. Correction of the finite size error for the chloride ion charge annihilation free energy. The charge annihilation free energy exhibits a significant size dependent trend (orange line), which can be corrected for (blue line) such that there is no longer any size dependence. The final chloride ion charge annihilation free energy is taken as the average of the corrected free energy from box length 3 nm to 10 nm (green line).

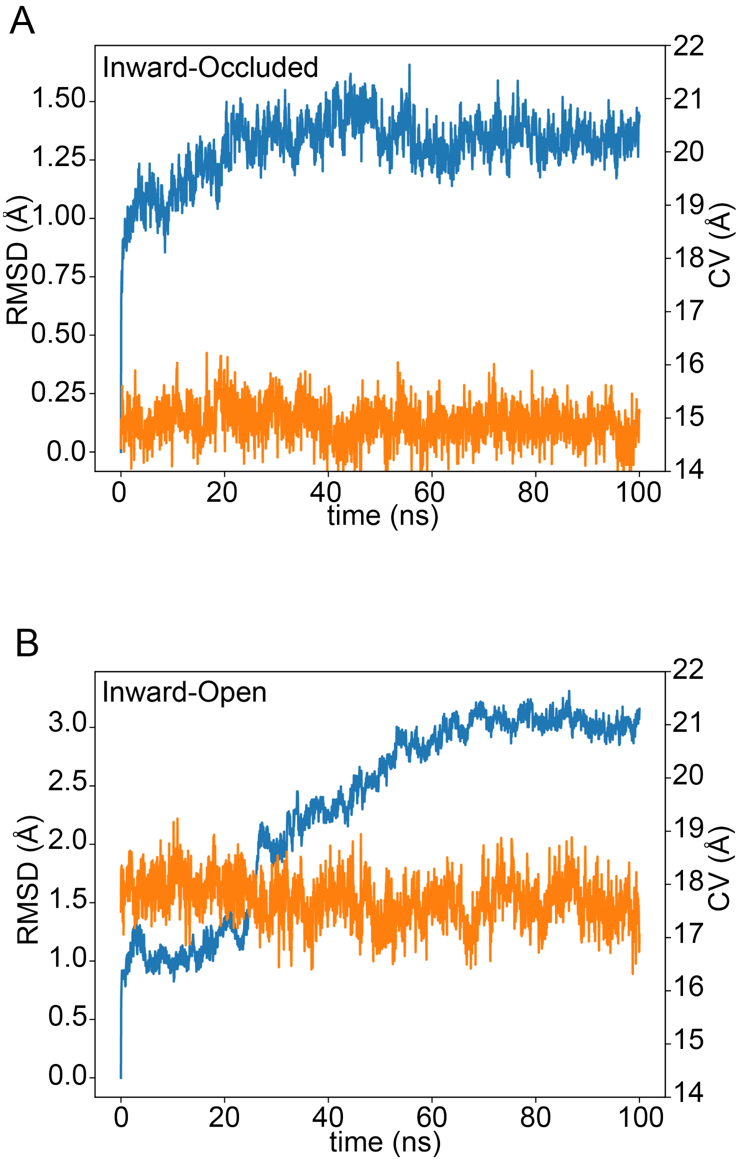

Figure S4. Dynamic behaviour of the (A) unbiased simulation of the inward-occluded protonated protein in complex with alanine and (B) unbiased simulation of the inward-open deprotonated protein in complex with alanine. The root mean squared deviation (RMSD) of the Cα atoms with respect to the first simulation frame (blue line) as well as the collective variable (orange line) defined as the distance between the center of mass of residues 113 to 117 and the center of mass of residues 235 to 239.

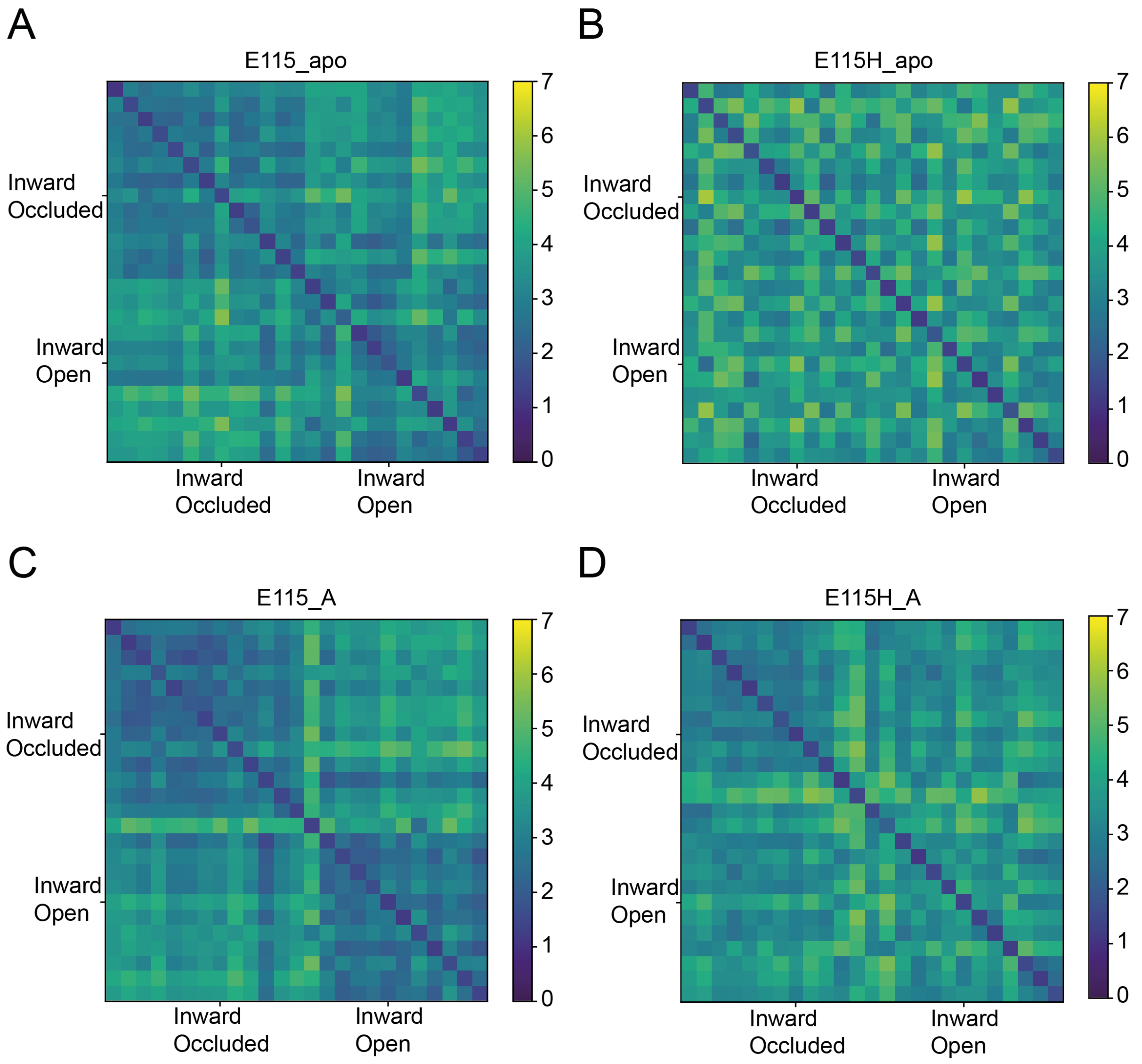

Figure S5. Average Cα RMSD of each CV window with respect to the end frame of all CV windows. Each pixel (*i*,*j*) is the average RMSD of trajectory in window *i* with respect to the last frame of window *j*.

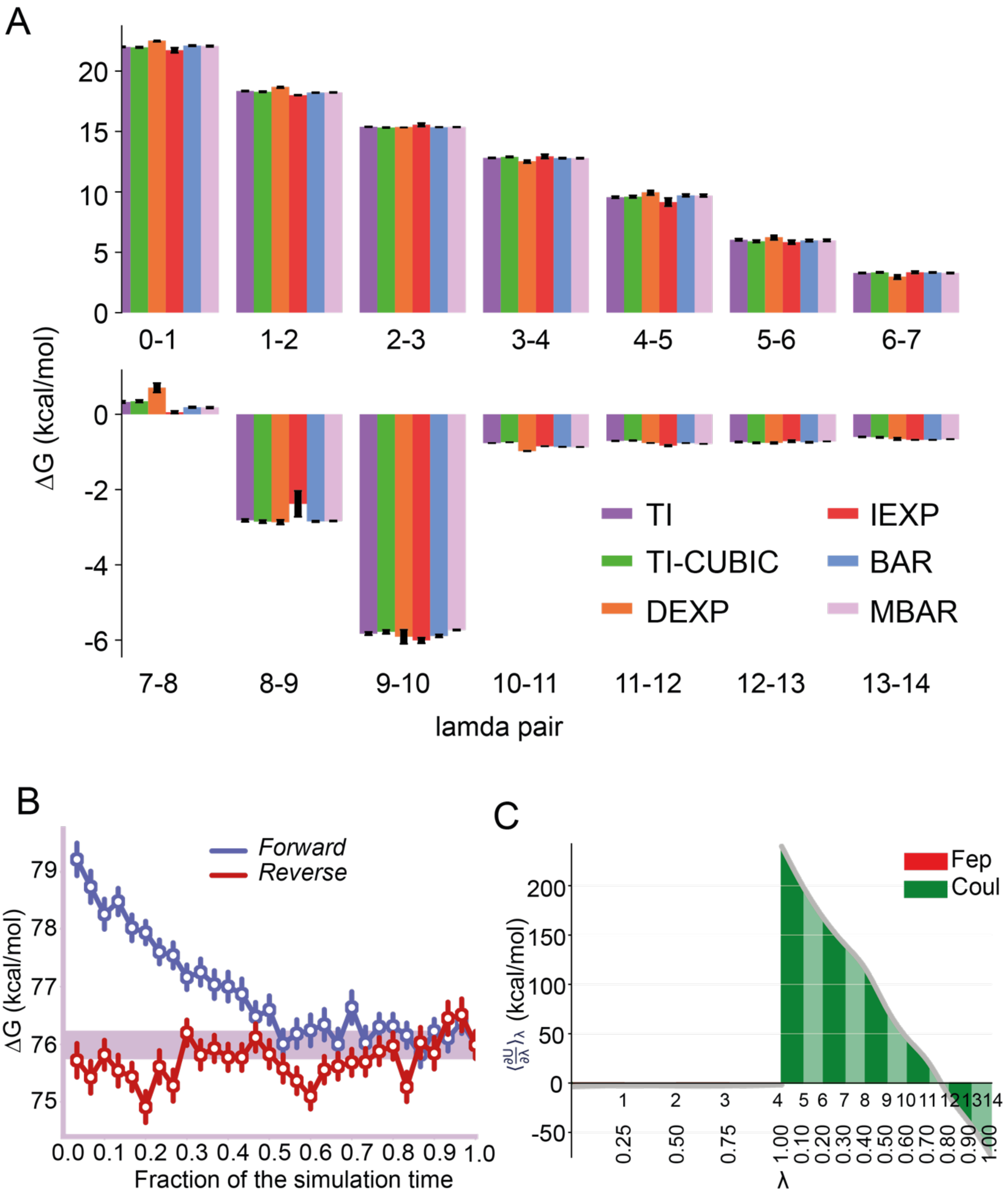

Figure S6. Convergence assessment measure for protonation of E115 in the apo inward occluded state. (**A**) Bar chart showing the free energy difference between adjacent lambda states evaluated via several methods along with the estimated error. (**B**). Free energy convergence in the sampling time axis. The free energy difference is shown in both forward series, where the value is the result of proportion of the data used (i.e. 0~10%, 0~20%) and in reverse fashion where the result is the reverse

proportion of the data (i.e. 90~100%, 80~100%). The simulation is stopped when the forward and reverse series converged to within 1 kcal/mol. (**C**). The gradient of the free energy with respect to lambda estimated for each lambda windows. The silver line is the interpolation of the gradient via the cubic spline and the area under the curve is the free energy integrated along the lambda axis. Coul indicates the free energy difference of alchemically changing the charge of the residue from protonated glutamate to deprotonated glutamate, while fep indicates the free energy change of the bonded, angle and dihedral terms as well as the annihilation of the vdw of the extra proton.

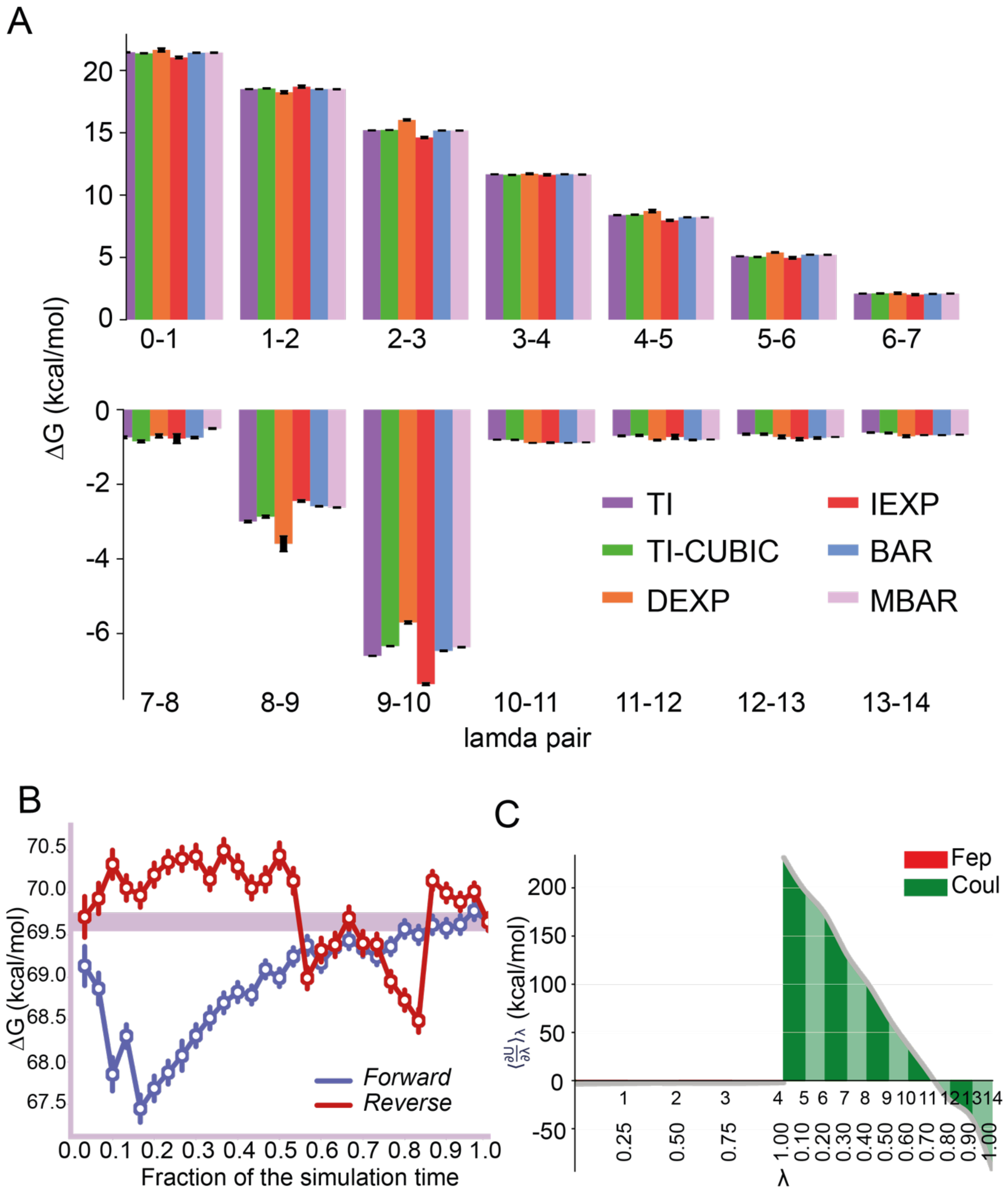

Figure S7. Convergence assessment measure for protonation of E115 in the apo inward open state. Panels **A-C** are as for Figure S5.

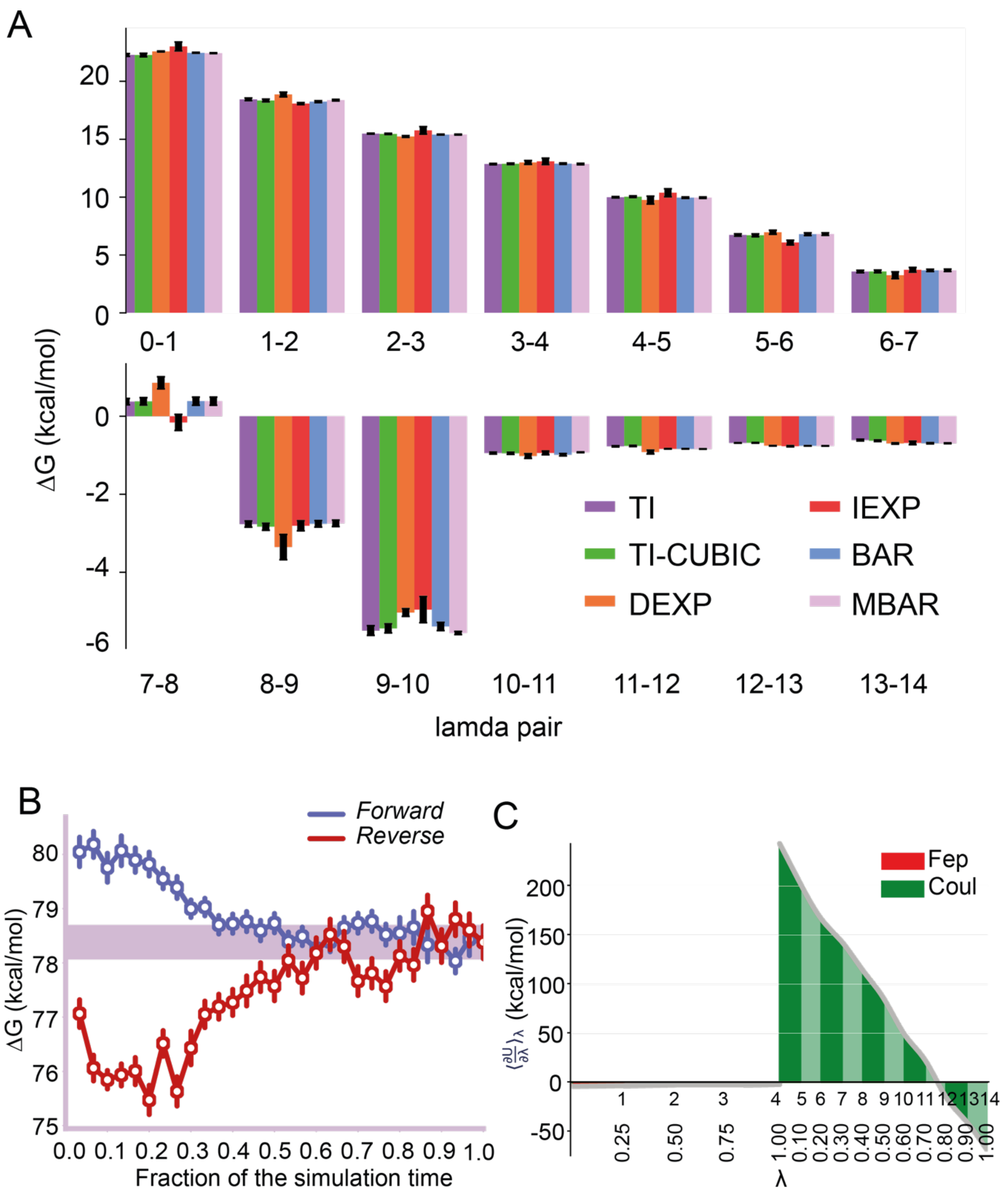

Figure S8. Convergence assessment measure for protonation of E115 in the alanine bound inward occluded state. Panels **A-C** are as for Figure S5.

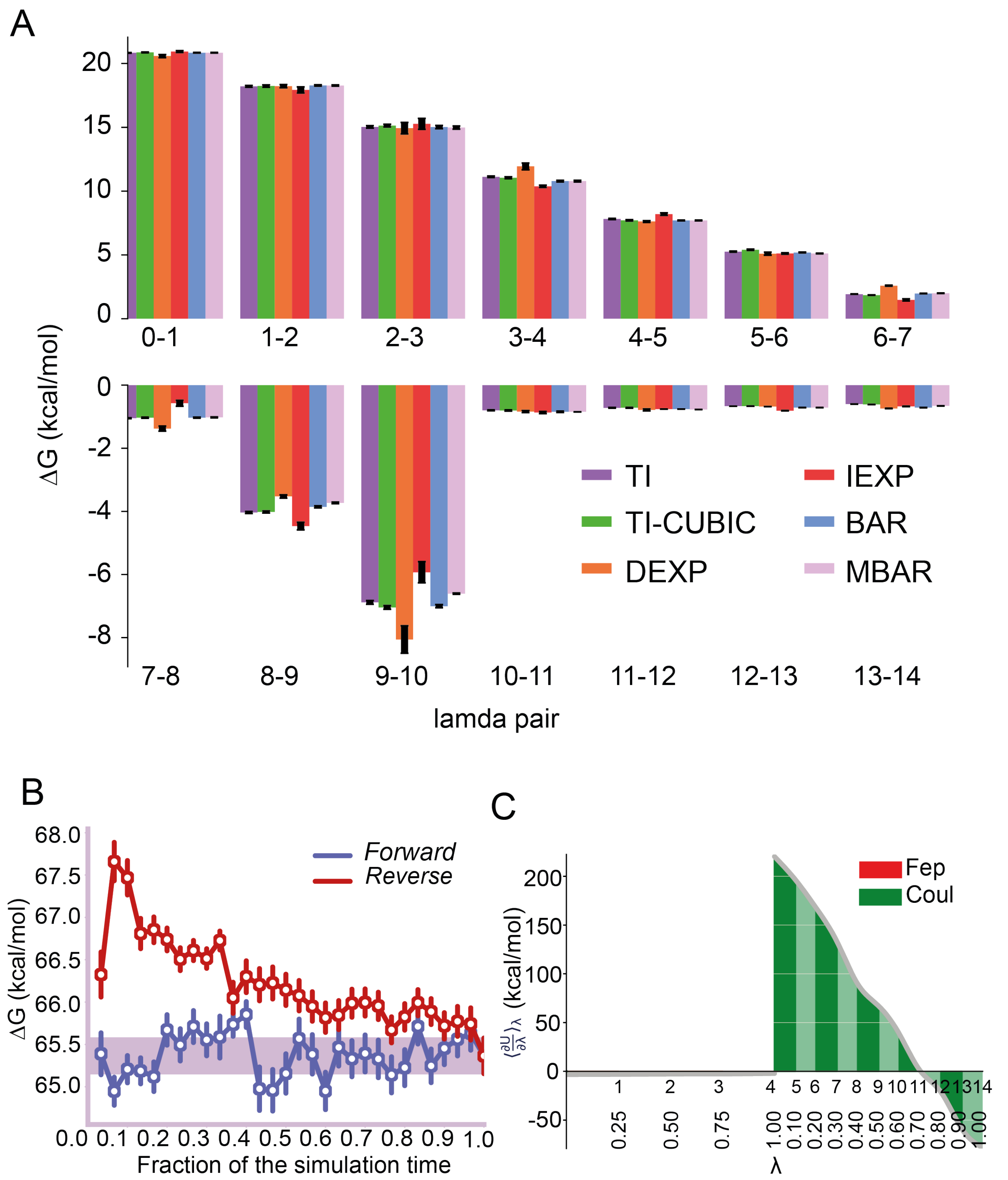

Figure S9. Convergence assessment measure for protonation of E115 in the alanine bound inward open state. Panels **A-C** are as for Figure S5.

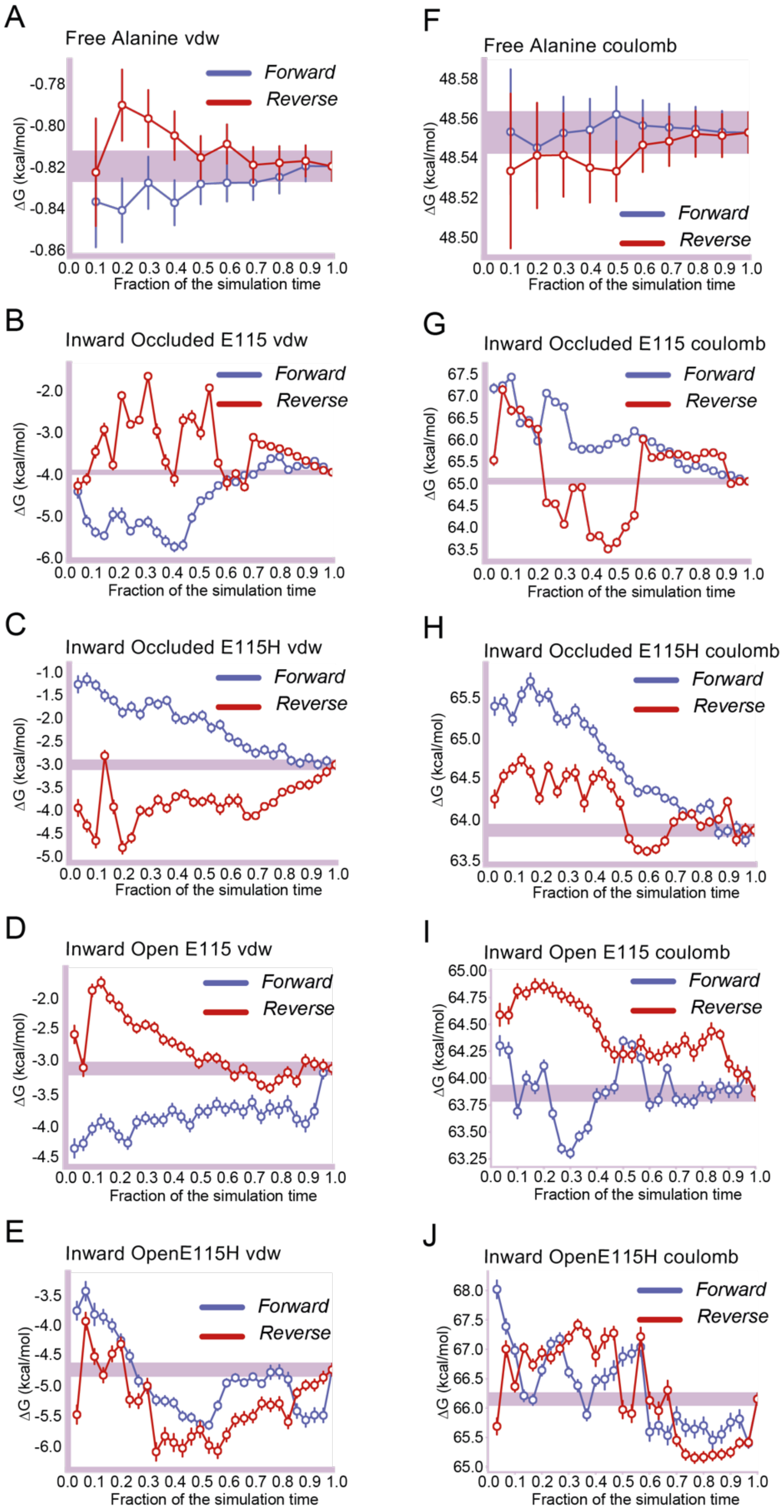

Figure S10. ΔG estimates as a fraction of simulation time. Panels **A-E** and **F-J** are vdw and coulombic contributions respectively. **A**, free alanine in solution; **B**, inward occluded E115; **C**, inward occluded E115H; **D**, inward open E115; **E**, inward open E115H; **F**, free alanine in solution; **G**, inward occluded E115; **H**, inward occluded E115H; **I**, inward open E115; and **J**, inward open E115H.
