# Supplementary material for: Proton Control of Transitions in an Amino Acid Transporter
Source: Biophys J. 2019 Aug 26;117(7):1342–51. doi: 10.1016/j.bpj.2019.07.056 (PMC6818167; doi:10.1016/j.bpj.2019.07.056)
Supplement: Document S2. Article plus Supporting Material [file mmc3.pdf]

Biophysical Journal
Article

# Proton Control of Transitions in an Amino Acid Transporter

Zhiyi Wu,[1] Irfan Alibay,[1] Simon Newstead,[1] and Philip C. Biggin[1,*]
[1]Department of Biochemistry, University of Oxford, Oxford, United Kingdom

ABSTRACT Amino acid transport into the cell is often coupled to the proton electrochemical gradient, as found in the solute carrier 36 family of proton-coupled amino acid transporters. Although no structure of a human proton-coupled amino acid transporter exists, the crystal structure of a related homolog from bacteria, GkApcT, has recently been solved in an inward-occluded state and allows an opportunity to examine how protons are coupled to amino acid transport. Our working hypothesis is that release of the amino acid substrate is facilitated by the deprotonation of a key glutamate residue (E115) located at the bottom of the binding pocket, which forms part of the intracellular gate, allowing the protein to transition from an inward-occluded to an inward-open conformation. During unbiased molecular dynamics simulations, we observed a transition from the inward-occluded state captured in the crystal structure to a much more open state, which we consider likely to be representative of the inward-open state associated with substrate release. To explore this and the role of protons in these transitions, we have used umbrella sampling to demonstrate that the transition from inward occluded to inward open is more energetically favorable when E115 is deprotonated. That E115 is likely to be protonated in the inward-occluded state and deprotonated in the inward-open state is further confirmed via the use of absolute binding free energies. Finally, we also show, via the use of absolute binding free energy calculations, that the affinity of the protein for alanine is very similar regardless of either the conformational state or the protonation of E115, presumably reflecting the fact that all the key interactions are deep within the binding cavity. Together, our results give a detailed picture of the role of protons in driving one of the major transitions in this transporter.

SIGNIFICANCE For transporter proteins that utilize the proton gradient to drive the uptake of solutes, the precise mechanistic details of proton coupling remain poorly understood. Structures can only infer the positions of protons. All-atom molecular dynamics simulations, however, are the ideal complementary tool. Here, we report extensive molecular dynamics simulations on GkApcT, a proton-coupled transporter. We observe a spontaneous transition from the crystallographically derived inward-occluded state to an inward-open state, which we then characterize with umbrella sampling and absolute binding free energy calculations. The results suggest that a conserved glutamate is protonated in the inward-occluded state, and subsequent deprotonation of this glutamate allows the transporter to move into the inward-open state, thus facilitating substrate release into the cell.

## INTRODUCTION

The intracellular concentrations of amino acids are regulated by various transporters that utilize different mechanisms. Transport can, in some cases, be coupled to another electrochemical gradient, such as sodium (1) or protons (2), which are used to drive the concentrative uptake of the amino acid into the cell. Proton-coupled amino acid transport systems, which include the solute carrier (SLC) 36 family of proton-coupled amino acid transporters, are also involved in mediating the export of amino acids from the lysosome in eukaryotic cells and, in certain instances, have been shown to regulate cellular metabolism through interactions with the mechanistic target of rapamycin (mTORC) kinase signaling complex (3,4). Some transporters can also act as facilitators, whereby the substrate is exchanged for its metabolized product (5) or another amino acid (6). This exchange activity can also be regulated by protons (pH) in which the transporter is only active in acidic pH (7). Although there have been many hypotheses put forward concerning the overall conformational cycle of secondary active transport proteins (8), the precise details of how the structure of these membrane proteins and their associated transport activity is regulated by protons remain incomplete.

*Submitted April 11, 2019, and accepted for publication July 30, 2019.*
*Correspondence: philip.biggin@bioch.ox.ac.uk
Editor: Michael Grabe.
https://doi.org/10.1016/j.bpj.2019.07.056
© 2019 Biophysical Society.
This is an open access article under the CC BY license (http://creativecommons.org/licenses/by/4.0/).

A proton-coupled amino acid transporter with the leucine transporter (LeuT) fold from *Geobacillus kaustophilus* (GkApcT) was recently solved in an inward-occluded state (9). GkApcT is distantly related to both the SLC7 family of cationic amino acid transporters and the SLC36 family of proton-coupled amino acid transporters. As such, it serves as a useful model system to understand how protons can be used to drive the transport of amino acids across cellular membranes. Like other members of the amino acid polyamine cation (APC) transporter superfamily, the structural fold of GkApcT is comprised of an inverted repeat of two five-helix bundles, with two additional helices in the C-terminal (Fig. 1 *A*). The overall transport process (9) is outlined in Fig. 1 *B*. The binding of the substrate to the outward-open state leads to the closure of the extracellular gate, giving rise to the outward-occluded state. The transporter then enters a state in which the extracellular gate is firmly closed, and the intracellular gate is primed to open. This state is termed the inward-occluded state and is followed by the opening of the intracellular gate and the release of the substrate, whereby the transporter is in the inward-open state, and the intracellular gate is fully open.

The crystal structure of GkApcT suggested that a glutamate residue (E115) within transmembrane helix (TM) 3, located at the intracellular side of the binding pocket, might be the key to the opening of the transporter toward the interior of the cell and the ejection of substrate. The intracellular gate, which sits underneath the amino acid binding pocket, also contains an aspartate residue, located on TM 6 (D237), which appears to stabilize the closed state of the

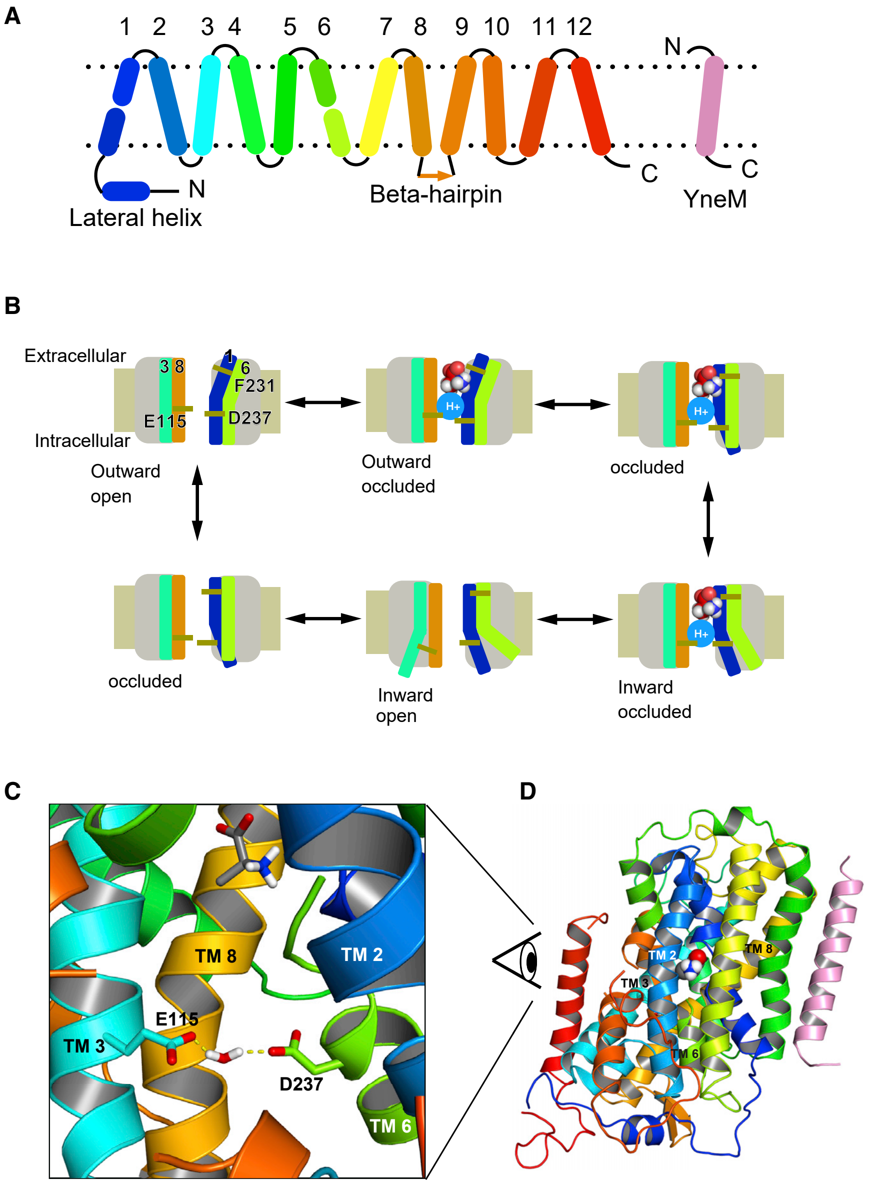


FIGURE 1 Structure and mechanism of GkApcT. (*A*) Shown is a schematic diagram of the GkApcT fold, in which the first five helices are an inverted repeat of the next five helices, similar to LeuT. The YneM helix (*pink*) is an additional subunit present in the crystal structure and included in the simulations presented here. (*B*) Mechanisms of the proton coupled transport cycle are as follows: outward-open state, outward-occluded state with substrate and proton bound, occluded state, inward-occluded state with substrate and proton ready to leave, inward-open state, and apo occluded state. (*C*) GkApcT in the inward-occluded state in complex with alanine is shown. The substrate alanine is drawn using the space fill representation. The two key residues gating the intracellular gate are E115 from TM 3 (*aqua stick*) and D237 from TM6 (*green*). The water molecule is not directly observed in the crystal but appears during the molecular dynamics simulations. (*D*) Shown is a zoomed-out view of GkApcT contextualizing the view in (*C*). To see this figure in color, go online.

intracellular gate by forming an interaction with E115 through a bridging water molecule (Fig. 1 *C*).

The role of protons in the transport mechanism of several transporters appears to control the formation and breaking of conserved salt bridge interactions between the extracellular and intracellular gating helices (10,11). We previously hypothesized (9) that the location of E115 and D237 in the intracellular gate might be the site of proton control in GkApcT. The close proximity of E115 to D237 in combination with a calculated pKa (via PROPKA (12)) value of 8.22 for E115 suggested this side chain was protonated in the crystal structure. Deprotonation via the intracellular side of the membrane should result in the repulsion of E115 from D237, driving the opening of the intracellular gate and the release of the bound amino acid.

To explore this further and evaluate this hypothesis, we have used appropriate computational methods (13) to examine the protonation of E115 and the influence this has on the free energy landscape of the inward-occluded to inward-open transition. In unbiased molecular dynamics (MD) simulations with E115 deprotonated, we were able to observe a spontaneous transition from the inward-occluded state to a new conformational state that is consistent with the properties expected for an inward-open state, including a clear exit path for the bound amino acid. We used this as the basis for the potential of mean force (PMF) and absolute binding free energy (ABFE) calculations to characterize the free energy landscape of the inward-occluded to inward-open transition.

Our results support a mechanism whereby deprotonation of E115 is necessary and sufficient to drive the transition to the inward-open state, thus enabling the release of the amino acid into the interior of the cell. The likely state of E115 protonation in either the inward-occluded or inward-open conformation is further confirmed by ABFE calculations and suggests that the ligand does not strongly influence this preference. Finally, ABFE simulations of alanine in the binding pocket in various states suggest that the affinity of the protein for alanine is not substantially influenced by protonation. Our results thus provide a detailed picture of the energetic landscape for the transition from the inward-occluded to inward-open state in GkApcT and a framework for understanding related mechanisms within the SLC36 family of proton-coupled transporters in mammalian cells.

## METHODS

### System preparation

The structure of the M321S mutant in complex with arginine (6F34) was used (9). Studies were performed on this mutant to allow a comparison with assay data in future work and to allow for the study of arginine in the future, something that is not possible with the wild-type transporter. In this study, however, we focus on alanine binding. The binding pose of alanine in the M321S mutant was generated by truncating the arginine side chain atoms beyond the C$\beta$ atom. Comparison with wild-type simulations suggested that the mutation has little effect on binding conformation (see Fig. S1). All organic molecules except cholesterol were removed, and missing residues were modeled using Modeler v9.20 (14). Briefly, the modeling procedure involved generating 1000 models, and the one with the best QMEANDisCo (QMEAN with distance constraints) score (15) was selected as the final model. During the equilibration phase, TIP3P water was found to sample the positions of all except one crystal water. This crystal water was buried in the pocket formed by the substrate and the transporter, bridging the C-terminus of the ligand to the protein. Thus, this crystal water was added explicitly as we considered it might be important for the recognition of the ligand by the protein.

### Bilayer setup

The GkApcT, YneM helix, and cholesterol obtained from x-ray crystallography were converted to a coarse-grained (CG) representation. The conversion was performed using the MARTINI v2.2 CG force field (16), in which four atomistic particles are represented as one "bead." The self-assembly process was initiated by adding 500 1-palmitoyl-2-oleoylphosphatidylcholine lipid molecules to the simulation box. CG simulation was then performed for 25 ns for the lipids to form a bilayer around the protein.

The 1-palmitoyl-2-oleoylphosphatidylcholine bilayer was converted to a mixture of 1-palmitoyl-2-oleoyl phosphatidylethanolamine (POPE) and 1-palmitoyl-2-oleoyl-*sn*-glycero-3-phosphoglycerol (POPG) in a 3:1 ratio to match the experimental conditions. A box with protein in its center that was twice as large as the bounding box of protein in all *x*, *y*, and *z* dimensions was sculpted from the CG simulation box. All the lipids outside this box were deleted. A 1-$\mu$s equilibration run was performed to allow POPE and POPG to equilibrate, and the final frame was converted to atomistic resolution using the Backward tool from the MARTINI toolkit (17).

The crystallographic coordinates of the GkApcT, YneM helix, cholesterol, and substrate arginine or alanine were superimposed to the backward-mapped POPE/POPG bilayer to replace their CG counterpart. The steric clashes between the POPE/POPG lipid and the protein were resolved using the InflateGro methodology (18). The resulting bilayer system was solvated with TIP3P water (19) in a cubic box with periodic boundary conditions and neutralized with NaCl to reach a final ionic concentration of 150 mM. The resulting atomistic system was energy minimized using the steepest descents method and allowed to equilibrate for 25 ns in NPT ensemble with positional restraints applied to GkApcT, YneM helix, and cholesterol.

### All-atom MD

Two replicates of 500-ns simulation were performed with E115 protonated and with E115 deprotonated and with alanine and arginine present. The AMBER ff99SB-ILDN force field (20) was used to describe the protein, and the Slipids force field (21) was used for the lipid molecules. The topology files for the zwitterions arginine and alanine were obtained from the AMBER parameter database (22). Protonation states of E115 were assigned using the pdb2gmx tool from GROMACS.

The integration timestep was 2 fs. The LINCS algorithm (23) was used to constrain H-bond lengths, and the long-range electrostatic interactions were calculated with the particle mesh Ewald method (24,25). The system was heated in NVT ensemble with V-rescale thermostat for 200 ps to reach a final temperature of 310° K. The system was then equilibrated in NPT ensemble for 5 ns with Nose-Hoover thermostat at 310° K and the Berendsen barostat (26) to keep pressure at one bar. During the equilibration phase, all heavy atoms were position restrained at 1000 kJ/mol/nm$^2$. The production runs were performed under the NPT ensemble, and the temperature was kept at 310° K using the Nose-Hoover thermostat (27) and the pressure at one bar using the Parrinello-Rahman barostat (28).

## Parameterization of protonated glutamate

As noted by Plamen Dobrev et al. (29), the rotation potential of the carboxyl H atom in Amber99SB-ILDN force field favors the syn orientation compared with the anti. Indeed, our preliminary simulations using the default parameters confirmed this observation, which is also inconsistent with the potential surface obtained from quantum mechanics (QM) calculations (Fig. S2). Thus, we have reparameterized the C-C-O-H dihedral such that the potential energy in TIP3P matches calculations performed at the HF/6-31G(d) level of treatment with a polarizable continuum model water solvent (using Gaussian 09).

We then reparameterized the glutamate using a glutamate residue with the N- and C-termini capped with acetyl and N-methyl groups, respectively, and solvated with TIP3P water in a triclinic box with 4-nm edge lengths. A sodium ion was also added to the box to neutralize the system charge. To minimize interactions between the glutamate residue and the sodium ion, the sodium ion was positionally restrained to the corner of the box, whereas the C$\alpha$ of the glutamate was restrained to the center of the box. An initial dihedral scan composed of 72 simulations with 5° steps, each with the C-C-O-H dihedral restrained at 3000 kJ/mol/rad, were performed with the C-C-O-H dihedral potential set to zero kcal/mol. Because of the instantaneous fluctuation of the water molecules, the potential energy of the system is taken as the average potential of the system across a 100-ns simulation. The resulting potential was fitted with a three-term Ryckaert-Bellemans potential to match the same dihedral potential surface obtained via QM (see Fig. S2). The dihedral scan was computed again with the new fitted Ryckaert-Bellemans potential to check that the result is consistent with the QM dihedral potential surface. Parameters for this refitted protonated glutamate residue can be found in Fig. S2.

## Umbrella sampling simulations and the PMF calculations

The collective variable used as our generalized coordinate for umbrella sampling was defined as the distance between E115 and D237. The coordinate of E115 was defined as the geometric center of the $\alpha$ carbons of residue number 113–117. The coordinate of D237 was defined as the geometric center of the $\alpha$ carbons of residue numbers 235–239. A total of 25 uniformly distributed windows were created along the 13–19 Å distance with a width of 0.25 Å. The initial frames for umbrella sampling were extracted from the unbiased simulation of GkApcT (M321S mutant) in complex with alanine with E115 deprotonated. The frame that was closest to the specified collective variable (CV) for each window was chosen as the initial frame for that window.

The umbrella sampling was performed in GROMACS 5.1.2 patched with the PLUMED library v2.3 (30). The bias applied to the collective variables was 1000 kJ/mol. The PMF profiles were generated using the weighted histogram analysis method as implemented in the WHAM tool by A. Grossfield (31). The error estimation is done in block analysis fashion, whereas WHAM analysis is performed with the last 50, 40, 30, 20, and 10% of the data. The error of the estimates is reported as the SD of these five values.

## Alanine ABFE calculations

Calculations were performed in GROMACS 2018.1 patched with the PLUMED library v2.4 (30) and followed a standard alchemical free energy cycle as previously described by us (32,33). The partial charges were annihilated over uniformly distributed windows with $\Delta\lambda = 0.1$, whereas 15 nonuniformly distributed $\lambda$ values (0.05, 0.1, 0.2, 0.3, 0.4, 0.5, 0.6, 0.65, 0.7, 0.75, 0.8, 0.85, 0.9, 0.95, and 1.0) were used for the decoupling of van der Waals interactions. Similarly, to restrain the substrate to the protein, 10 nonuniformly distributed $\lambda$ values (0.01, 0.025, 0.05, 0.075, 0.1, 0.2, 0.35, 0.5, 0.75, and 1.0) were used. Thus, a total of 36 windows for the substrate and protein complex simulation and ligand simulation were employed. A timestep of 2 fs was used for the restraint and charge annihilation free energy steps. As the addition of Boresch restraint introduced a high frequency motion to the system, which is more predominate near the end of the decoupling process, a 1-fs timestep was used for the decoupling of the ligand near the start and a 0.5-fs timestep was used for windows near the end of the process. The conformations of the protein were restrained by using a harmonic restraint of 1000 kJ/mol/nm$^2$ on the collective variable as described above for the umbrella sampling simulations. The value of the collective variable for each conformation were defined as the distance of the lowest point in the PMF for that corresponding protonation state. After equilibration, dH/d$\lambda$ data were collected from 30 ns of production runs. The relative position and orientation of the substrate to the protein were restrained using six atoms from the protein backbone (T43:CA, T43:C, G44:N) and the alanine backbone (A:C, A:CA, A:N) in the form of one distance (G44:N, A:C), two angles (T43:C, G44:N, A:C and G44:N, A:C, A:CA), and three dihedrals (T43:CA, T43:C, G44:N, A:C; T43:C, G44:N, A:C, A:CA and G44:N, A:C, A:CA, A:N); restraints were applied, and the equilibrium values were taken from the ensemble average of a 100-ns unbiased simulation at each conditions. The restraint free energy was evaluated analytically (34). The collected dh/d$\lambda$ were processed using the alchemical analysis Python script (35) using the multistate Bennet acceptance ratio (MBAR) method, and the overall error was estimated using error propagation on the MBAR error.

## Protonation free energy calculations

Calculations were carried out in GROMACS 2018.1 in a similar manner to the ABFE calculations, in which the protonated glutamate was alchemically transformed to deprotonated glutamate. The alchemical transformation is done in a single topology manner with a topology file generated using pmx (36,37). The alchemical charge transformation is done over 11 windows from protonated glutamate to deprotonated glutamate with $\Delta\lambda = 0.1$. Five $\lambda$ windows ($\Delta\lambda = 0.25$) were also used to transform the bond length, angle, and van der Waals all at the same time. Thus, a total of 16 windows were run for 30 ns each, and the result was analyzed using the alchemical analysis script. To balance out the finite-size problem introduced by the non-neutral box at the end state, a chloride ion was alchemically transformed at the same time to keep the box neutral. To prevent interactions between the glutamate and chloride ion, the chloride ion is position restrained to the corner of the box, ensuring an 80 Å separation between the two alchemically transform objects.

The free energy of annihilation of the charge of the chloride ion were deducted from the final result. The charge annihilation free energy was initially obtained from simulating a chloride ion in a cubic box of water. The charge of the chloride ion was annihilated with $\Delta\lambda = 0.1$. The simulation setup is the same as all other free energy calculations. The Rocklin correction (38) was applied to correct for the size-dependent finite-size error. Different box sizes with edge lengths ranging from 30 to 100 Å with a step of 10 Å were tested to ensure the final corrected charge annihilation free energy is size independent (see Fig. S3). For these eight box sizes, three replicates were performed, and the final chloride ion charge annihilation free energy is taken as the average of the 8 × 3 trials.

# RESULTS

## Protonation of E115 stabilizes the inward-occluded state of the transporter

GkApcT functions to couple amino acid uptake to the inwardly directed proton electrochemical gradient (9). The crystal structure was captured in a so-called inward-occluded state, whereby the extracellular gate was tightly

sealed, and the intracellular gate was partially open but occluded enough to prevent the bound amino acid from accessing the intracellular solution. The transition from the inward-occluded state to the fully inward-open state was postulated to involve an increase in the separation between TMs 3 and 6 (Fig. 1 *B*). Given the location of E115 on TM3, and its unusual pKa of 8.2 compared to 4.38 for D237 (calculated by PROPKA3 (12)), we postulated the conformation of the intracellular gate is likely dependent on the protonation state of this side chain (9).

To investigate this in the first instance, we performed a series of unbiased 500-ns MD simulations of GkApcT in a POPE/POPG (3:1 ratio) lipid bilayer (Fig. 2) with E115 in different protonation states and in the presence and absence of an amino acid (either alanine or arginine—see Methods). During one of the simulations with E115 deprotonated, we observed a significant expansion of the binding cavity (Fig. 2, *B* and *C*) along with an increase in the distance between TM3 and TM6 at the intracellular end of the helices, resulting in a state that would be consistent with an inward-open conformation. In contrast, this movement was not observed when E115 remained protonated.

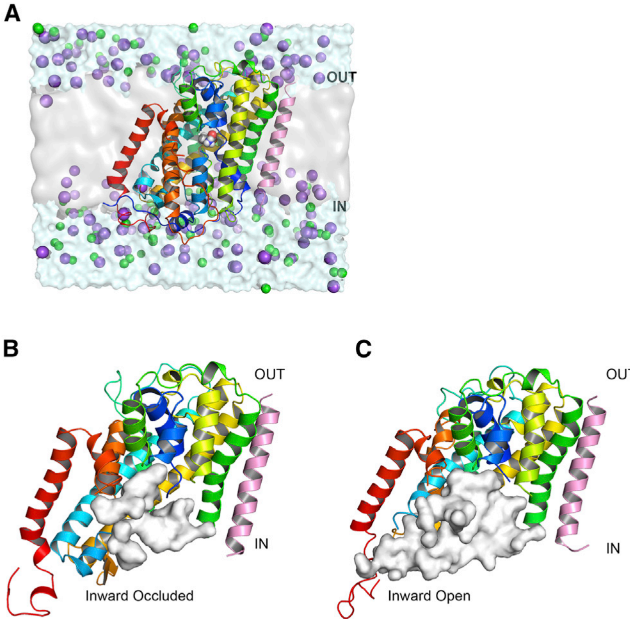


FIGURE 2 Unbiased molecular dynamics simulations of GkApcT in a lipid bilayer. (*A*) Shown is a representative snapshot of the simulation system—GkApcT embedded in a POPE/POPG bilayer with alanine in the binding pocket. Sodium ions are represented as purple spheres, and chloride ions are represented as green spheres. (*B*) GkApcT in inward-occluded conformation and (*C*) inward-open conformation is shown. The conformational change from inward occluded to inward open results in an expansion of the binding cavity as represented by the white surface. In the inward-open conformation, the binding cavity extends to the bulk of intracellular space, giving rise to a potential substrate exit pathway. Some sections of helices are not displayed to help visualize the extent of the cavity. To see this figure in color, go online.

## Transition to an inward-open state following deprotonation of E115

To characterize the new conformational state and to establish whether indeed it could be considered a possible inward-open state, we first pulled the alanine out of the binding pocket using steered MD with a moving restraint that increased the distance between the binding pocket, defined as the center of mass of the C$\alpha$ of residues I40, T43, G44, F231, and I234 (i.e., the residues that make hydrogen bonds to the bound alanine in the binding site) and the C$\alpha$ atom of the alanine. Fig. 3 *A* shows the profile of the work done for five such pulling simulations as a function of this vector. The plateaus (at 60 ns and beyond in all simulations) correspond to the point in time when there are no hydrogen bond interactions between the alanine and the residues of the binding site, and the ligand exhibits random motion within the cavity. Although these profiles are far from converged, we were ultimately focused on whether there were further impediments to access to the bulk solution beyond that of the binding site. These simple pulling simulations show there are none, and the amino acid remains bound only through the interactions with the binding site residues.

To explore this further, we took one snapshot from the end of one of the pulling simulations, which we considered was fully dissociated from the binding pocket, and performed free MD from this starting point. After 180 ns of simulation time, we observed the spontaneous rebinding of alanine to the binding pocket (Video S1). The main barrier to the exit of alanine from the inward-occluded state is a hydrophobic gate formed by A238 on TM6 and V328 on TM8. In the crystal structure, these act (Fig. 3 *B*) to occlude access to the bulk solution. In the inward-open state, however, the separation of these helices creates a large enough space to allow alanine to move quite freely (the minimal separation is $\sim$6.5 Å between atoms A238 and V328), as is shown in Fig. 3 *C* alongside a timeline trace of the path of alanine as it moves back into the binding pocket. Closer inspection of the binding event revealed that the binding mode is practically identical to that observed in the crystal structure (Fig. 3, *D* and *E*) with a heavy atom root mean-squared deviation (RMSD) of 0.8 $\pm$ 0.2 Å over the last 35 ns.

Thus, this observation and the steered MD profiles confirm that there is no steric barrier to ligand egress from the binding site, and thus, the final state sampled in our simulations is likely to be reflective of a genuine inward-open state.

## PMF calculations identify E115 as an important part of the proton coupling mechanism

Satisfied that the opening we observed represented a plausible inward-open state, we decided to explore the possible transition between these two states further. Given the

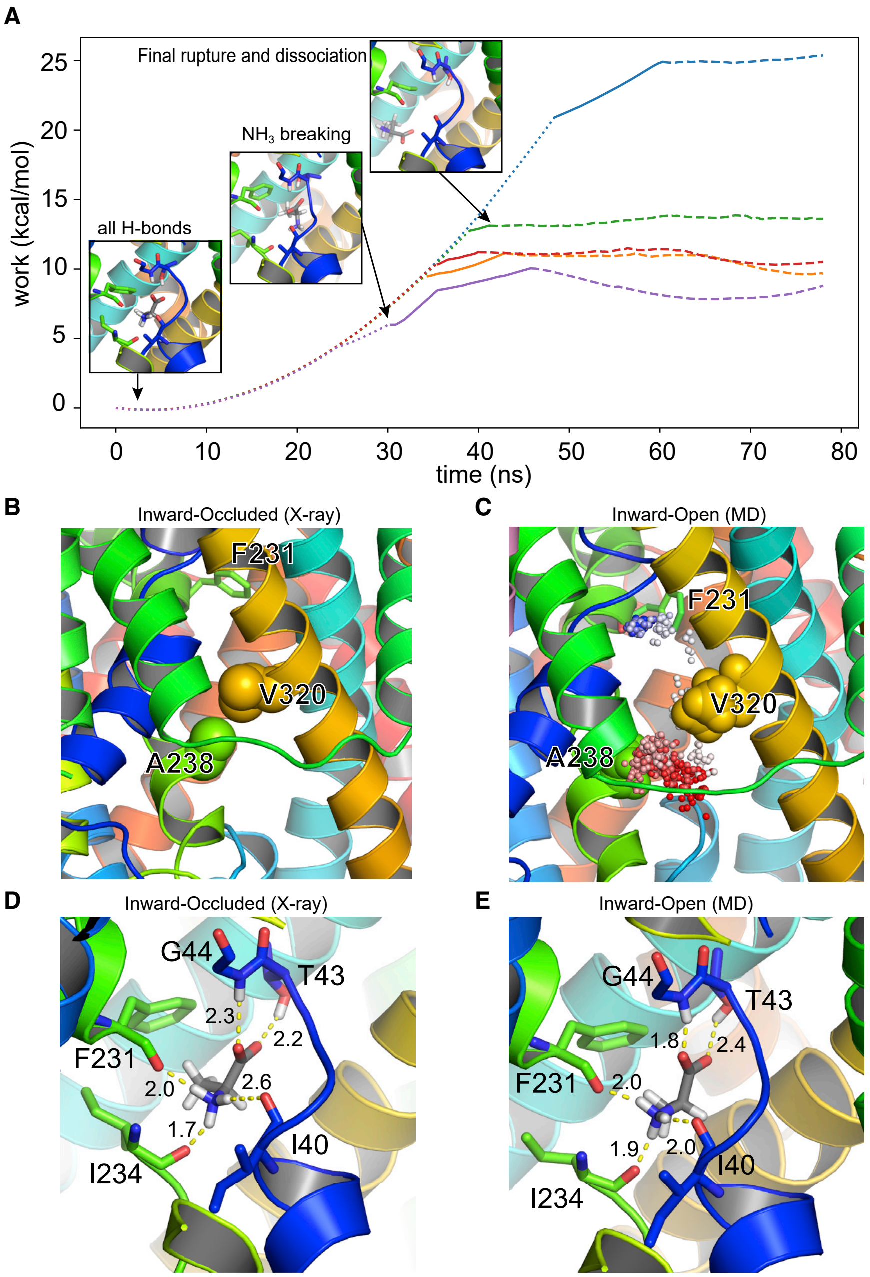


FIGURE 3 (*A*) Work performed as function of time in the pulling simulation. The dotted section of the line represents the state in which alanine is fully coordinated in the binding pocket. The solid section is where the interaction between the amine group of alanine and the protein is broken, whereas the interaction between the carboxyl group and protein is still intact. The dashed line represents the state in which alanine is fully dissociated from the binding pocket. Snapshots give an indication of the nature of the binding event at the various transition points. The plateaus reflect the alanine diffusing randomly within the cavity away from the binding site. (*B*) shows the gate in the inward-occluded state formed by A238 and V320 drawn as spheres (note no hydrogens are present in the crystal structure). (*C*). Shown is the path (as shown as the *colored dots* from *red* to *blue* as a function of time) of the alanine in an unbiased simulation that show rebinding to the binding site (indicated by the F231 residue) during the inward-open state. (*D* and *E*) show a comparison of alanine binding to the inward-occluded and inward-open states, respectively. To see this figure in color, go online.

potential role of protonation on E115, we decided to first use umbrella sampling (see Methods) on the various systems (E115 protonated/deprotonated, alanine present/absent) to map out the energy landscape of this conformational change. We used snapshots from the transition observed in the unbiased simulations as the starting points and constructed a collective variable defined as the distance between the center of mass of the C$\alpha$ atoms of residues 113–117 on TM3 and the center of mass of the C$\alpha$ atoms of residues 235–239 on TM6 because that distance appeared to correlate well with the opening of the cavity. PMF profiles were calculated for this collective variable (see Methods). Fig. 4 summarizes the calculations for all systems.

Apart from the deprotonated E115 with alanine bound system (Fig. 4 *E*), the PMF profiles show a single well, suggesting that this collective variable is not, on its own, able to fully describe the energy landscape separating the two putative states identified from the free simulations described above. This is, perhaps, to be expected, given the presence of the proton is known to dramatically shift the equilibrium between conformational states. Thus, this particular collective variable can only be used as an approximate indicator of the energetic landscape. That this is a reasonable stance to adopt is supported by an examination of the conformational dynamics of unbiased simulations (100 ns) starting from snapshots with the CV distances coincident with the

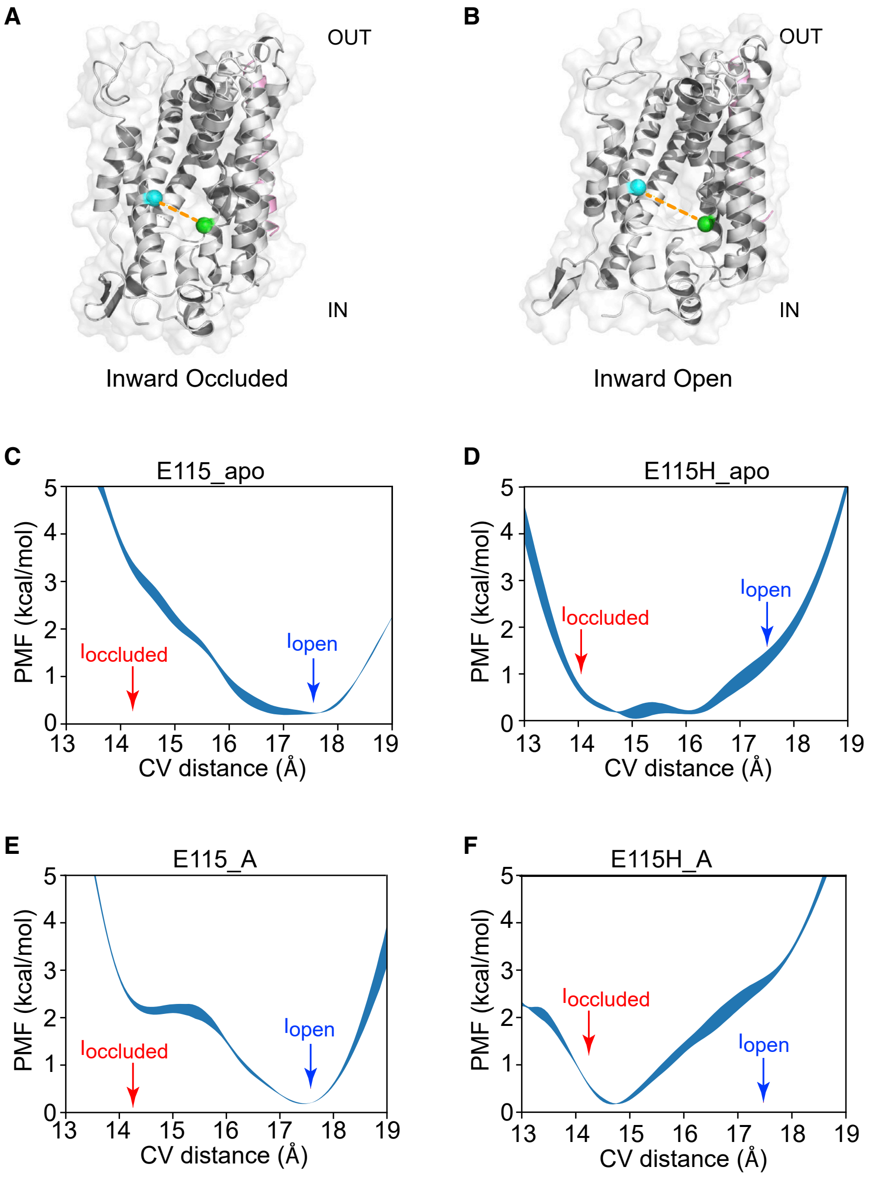


FIGURE 4 (*A* and *B*) The space between two green spheres represents E115 and D237. The distance between the two spheres increases as GkApcT transitions from inward occluded (*A*) to inward open (*B*). This distance formed the basis of the collective variable used in later umbrella sampling calculations. (*C–F*) Shown is the PMF calculation using umbrella sampling. The $x$ axis is the collective variable, which is the distance between the center of mass of the C$\alpha$ atoms of residues 113–117 and the center of mass of the C$\alpha$ atoms of residues 235–237. The relative locations of the inward-occluded and inward-open state are annotated by red and blue arrows, respectively. PMF profiles are shown for (*C*) apo with deprotonated E115, (*D*) apo with protonated E115, (*E*) deprotonated with alanine in the binding site, and (*F*) protonated E115 with alanine bound. The width of the PMF profile represents the error associated with the potential at that point, calculated using block analysis as described in the Methods. To see this figure in color, go online.

inward-occluded or the (putative) inward-open states (Fig. S4). In both cases, the CV distances exhibit small fluctuations (1–1.5 Å) around their initial values. The corresponding heavy atom RMSD values also plateau at low levels (1.5 Å for the inward occluded and ~3.0 Å for the inward open). Thus, the protein does not exhibit large conformational changes in the free simulations, suggesting that the collective variable distance is in some way representing the conformational state of the protein as a whole.

In the PMF profiles, the appearance of a single well for the deprotonated E115 apo state (Fig. 4 *C*) suggests that deprotonated E115 stabilizes the inward-open state (or something close to it) and destabilizes the inward-occluded state. In comparison, the PMF exhibits a single, but broader, well when E115 is protonated in the apo state (Fig. 4 *D*). Compared to the apo PMFs (Fig. 4, *C* and *D*), the presence of alanine in the binding pocket only has a modest effect on the profiles (Fig. 4, *E* and *F*). In the deprotonated E115 with alanine bound profile (Fig. 4 *E*), the CV appears to capture the two states, with the inward-occluded state being metastable compared to the inward-open state, which is lower by ~2.2 kcal/mol (the potential difference in energy between the wells). The protonated E115 with alanine bound PMF gives a single steep-sided well. Plots of the average C$\alpha$ RMSD of each CV window with respect to the end frame of all CV windows are consistent with the shape of the profiles (Fig. S5). For example, that there are two metastable states for E115 with alanine is supported by the RMSD matrix (Fig. S5 *C*). Likewise, that the E115H apo PMF (Fig. 4 *D*) is a broad well is reflected in the RMSD matrix (Fig. S5 *B*) in which the RMSD values for each of the windows show a higher degree of difference.

Overall, although the CV does not provide a clean delineation of the two conformational states under the conditions explored, we are able to make the qualitative assessment that deprotonating E115 favors a conformation closer to the inward-open state and protonating E115 favors a conformation closer to the inward-occluded state.

## ABFEs confirm a role for protonation of E115 in stabilizing the inward-occluded state

Although the PMF calculations gave results consistent with current hypotheses on proton-amino acid transporters, we sought to independently verify the energetics via the use of ABFE. The standard thermodynamic cycle approach was used (see Methods; Fig. S6–S10; Tables 1 and 2). We first used ABFE calculations to calculate the free energy associated with protonating E115 (Table 1). The results suggest that protonation of E115 is favored in the inward-occluded state, completely consistent with the results obtained from the PMF calculations. These values can be directly converted (i.e., no standard volume or entropy corrections applied) to an equivalent "pseudo" pKa, which shows the expected trend.

We then used ABFE to compute the free energies of alanine binding to the different conformational states and different protonation states. Those results are summarized in Table 2. Interestingly, it can be seen that protonation of E115 does not change the affinity of alanine for the inward-occluded state. The affinity of alanine for the inward-open state is slightly reduced but only by 0.2 kcal/mol, too small to indicate any significant difference to how the amino acid is recognized within the transporter.

## DISCUSSION

MD simulation has emerged as a powerful tool to understand the role of ion binding to several members of the APC superfamily, including the sodium-coupled transporter LeuT (39,40). However, GkApcT is coupled to protons rather than sodium, and the question of how the same protein fold has evolved to couple to different ion gradients is ongoing within the transporter community (41). The observation that members of the SLC36 family of proton-coupled amino acid transporters regulate the mTORC complex and shuttle between the plasma membrane and lysosomal membranes, which both contain a pH gradient across them, further underscores the need to understand the role of protons in regulating transporter function within the cell.

MD simulations are an ideal tool to study this kind of problem, but one must be careful, especially when trying to interpret short timescale events occurring in the simulations and their relevance to longer timescale observables. Indeed, the implicit assumption of many MD studies that local conformational changes observed in short simulations can provide insight into global motions has recently been called into question (42). It is clear that very long simulation times are often required, as recently demonstrated for the semiSWEET transporter (43) or the GluT1 transporter (44). Indeed, to study the complete conformation cycle, it would seem likely that the approach of Markov state modeling might be the most appropriate as recently demonstrated with $PepT_{so}$ transporter (45). In this work, however, we were interested in fully characterizing only one part of the full transition pathway, facilitating the use of shorter timescales that enabled the application of more rigorous methods.

We first examined whether the deprotonation of E115 facilitates the opening of the intracellular gate in GkApcT. The PMF calculations are consistent with our earlier hypothesis (9) that deprotonation of E115 appears to favor the inward-open state. However, our results also show that this conformational change is necessary for the substrate to exit (or enter) the binding pocket from the interior of the cell in unbiased simulations. Although PMF calculations have been reported to obtain a reasonable estimate of unbinding (for example, xylopyranose from XylE (46)), we could not rule out alternative dissociation pathways and hence elected to use ABFE calculations to support our PMF observations. Using ABFE calculations, we have shown that protonation of E115 stabilizes the inward-occluded state and that this is the case regardless of the presence of ligand in the binding site. The ABFE calculations for alanine affinity were initially surprising to us in that they did not reveal weaker binding to the inward-open state as one might perhaps expect. However, when one considers that the key interactions between the protein and ligand are deep in the cavity and do not appear to change much between the inward-occluded and inward-open states (Fig. 3, *D* and *E*), then the lack of difference is less surprising. It was recently suggested that for LeuT, via the use of single-molecule fluorescence resonance energy transfer, partially open intermediates were associated with transport activity (47). We would suggest a note of caution here as although our inward-open state appears alluring, we do not know whether a more open (or different) inward-open state with more significant changes in the binding might be possible. Indeed, the PMF (Fig. 4) calculations alongside a comparison of the protein conformations (Fig. S4) suggest that the energetic landscape in this region is rather flat and that many conformations may exist that allow the substrate

**TABLE 1 Summary of Free Energy to Protonate the E115 Side Chain and the Corresponding pKa**

| | Inward Occluded | | Inward Open | |
|---|---|---|---|---|
| | $\Delta G$ | pKa[a] | $\Delta G$ | pKa[a] |
| Apo | −3.62 ± 0.2 | 9.55 ± 0.1 | +2.72 ± 0.1 | 5.08 ± 0.1 |
| Alanine bound | −6.01 ± 0.2 | 11.23 ± 0.1 | +7.01 ± 0.2 | 2.05 ± 0.1 |

Errors are estimated by MBAR under error propagation (see Methods). All energies are in kcal/mol.
[a]No corrections to the pKa beyond those already mentioned in Methods are applied.

**TABLE 2 Absolute Free Energies for Alanine**

| | Alanine in Water | Alanine in E115_Inward Occluded | Alanine in E115_Inward Open | Alanine in E115H_Inward Occluded | Alanine in E115H_Inward Open |
|---|---|---|---|---|---|
| $\Delta G$ | −47.73 ± 0.01 | −5.89 ± 0.04 | −5.55 ± 0.07 | −5.67 ± 0.08 | −5.27 ± 0.14 |

All energies in kcal/mol ± SD. Errors are estimated by MBAR under error propagation (see Methods).

to leave. Defining a simple distance-based collective variable that captures this may in fact not be possible and hinders the construction of a full thermodynamic cycle at this stage. The likely protonation states that are favored for each state are, however, supported by the ABFE calculations. However, the calculation of protonation states with a fixed charged model as we have performed here is well known to be problematic (48,49), and more work is clearly needed in this area. Nevertheless, what we are ultimately interested in here is the relative stability of different states rather than the absolute values.

Another question that remains at this point is where the proton moves to after it leaves E115. That is beyond the scope of the current work, and dealing with proton hopping in complex systems is far from trivial, although there is promising work in this direction (50). However, it is likely to involve water, and indeed, the role of water in driving conformational transitions is made very apparent here. Water penetration into the inward-occluded state will facilitate the deprotonation step and accelerate the probability of moving into the inward-open state. A similar role has been proposed on the basis of MD simulations for the DAT (51), SERT (52), the XylE (53), and LeuT transporters (54,55), whereby water penetration leads to the release of the Na2-bound $Na^{+}$, which in turn leads to destabilization of the substrate. It would be interesting to examine this further with constant pH simulations as has been demonstrated for the sodium-proton antiporter (56,57). Ideally, some of the aspects discussed above would also be validated experimentally, but this has proven particularly challenging for this system thus far, and so at this stage, we must content ourselves with the computational observations.

## CONCLUSION

We have provided mechanistic insight into the role of proton-coupled transport in GkApcT. Specifically, we have developed a model for how the proton and substrate are released from the inward-occluded state. Our results provide further evidence that deprotonation of E115 is associated with the opening of the intracellular gate as well as the transition from inward-occluded to inward-open state. This conformational change opens up the passage from the binding pocket to the intracellular space, allowing the release of the substrate. Importantly, E115 is conserved in the mammalian SLC36 homologs, suggesting that this residue is likely to form part of a more conserved mechanism for proton coupling within the APC superfamily.

## SUPPORTING MATERIAL

Supporting Material can be found online at https://doi.org/10.1016/j.bpj.2019.07.056.

## AUTHOR CONTRIBUTIONS

P.C.B. and S.N. designed the project. Z.W. performed the calculations. I.A. advised on PMF and free energy perturbation interpretation. I.A., Z.W., S.N., and P.C.B. analyzed the data. Z.W., S.N., and P.C.B. wrote the manuscript.

## ACKNOWLEDGMENTS

S.N. and Z.W. thank the Wellcome Trust (203741/Z/16/Z and 102890/Z/13/Z) for support. This project made use of time on JADE (EP/P020275/1) and ARCHER granted via the UK High-End Computing Consortium for Biomolecular Simulation (http://www.hecbiosim.ac.uk) supported by Engineering and Physical Sciences Research Council (EP/L000253/1).

## REFERENCES


1. Krishnamurthy, H., and E. Gouaux. 2012. X-ray structures of LeuT in substrate-free outward-open and apo inward-open states. *Nature.* 481:469–474.
2. Thwaites, D. T., and C. M. Anderson. 2011. The SLC36 family of proton-coupled amino acid transporters and their potential role in drug transport. *Br. J. Pharmacol.* 164:1802–1816.
3. Wolfson, R. L., and D. M. Sabatini. 2017. The dawn of the age of amino acid sensors for the mTORC1 pathway. *Cell Metab.* 26:301–309.
4. Heublein, S., S. Kazi, …, D. C. Goberdhan. 2010. Proton-assisted amino-acid transporters are conserved regulators of proliferation and amino-acid-dependent mTORC1 activation. *Oncogene.* 29:4068–4079.
5. Closs, E. I., J. P. Boissel, …, A. Rotmann. 2006. Structure and function of cationic amino acid transporters (CATs). *J. Membr. Biol.* 213:67–77.
6. Fotiadis, D., Y. Kanai, and M. Palacín. 2013. The SLC3 and SLC7 families of amino acid transporters. *Mol. Aspects Med.* 34:139–158.
7. Shaffer, P. L., A. Goehring, …, E. Gouaux. 2009. Structure and mechanism of a Na+-independent amino acid transporter. *Science.* 325:1010–1014.
8. Drew, D., and O. Boudker. 2016. Shared molecular mechanisms of membrane transporters. *Annu. Rev. Biochem.* 85:543–572.
9. Jungnickel, K. E. J., J. L. Parker, and S. Newstead. 2018. Structural basis for amino acid transport by the CAT family of SLC7 transporters. *Nat. Commun.* 9:550.
10. Coincon, M., P. Uzdavinys, …, D. Drew. 2016. Crystal structures reveal the molecular basis of ion translocation in sodium/proton antiporters. *Nat. Struct. Mol. Biol.* 23:248–255.
11. Newstead, S. 2015. Molecular insights into proton coupled peptide transport in the PTR family of oligopeptide transporters. *Biochim. Biophys. Acta.* 1850:488–499.
12. Olsson, M. H. M., C. R. Søndergaard, …, J. H. Jensen. 2011. PROPKA3: consistent treatment of internal and surface residues in empirical pKa predictions. *J. Chem. Theory Comput.* 7:525–537.

13. Pieńko, T., and J. Trylska. 2019. Computational methods used to explore transport events in biological systems. *J. Chem. Inf. Model.* 59:1772–1781.

14. Eswar, N., B. Webb, …, A. Sali. 2006. Comparative protein structure modeling using Modeller. *Curr. Protoc. Bioinformatics.* Chapter 5:Unit-5.6.

15. Waterhouse, A., M. Bertoni, …, T. Schwede. 2018. SWISS-MODEL: homology modelling of protein structures and complexes. *Nucleic Acids Res.* 46:W296–W303.

16. Marrink, S. J., H. J. Risselada, …, A. H. de Vries. 2007. The MARTINI force field: coarse grained model for biomolecular simulations. *J. Phys. Chem. B.* 111:7812–7824.

17. Wassenaar, T. A., K. Pluhackova, …, D. P. Tieleman. 2014. Going backward: a flexible geometric approach to reverse transformation from coarse grained to atomistic models. *J. Chem. Theory Comput.* 10:676–690.

18. Kandt, C., W. L. Ash, and D. P. Tieleman. 2007. Setting up and running molecular dynamics simulations of membrane proteins. *Methods.* 41:475–488.

19. Jorgensen, W. L., J. Chandresekhar, …, M. L. Klein. 1983. Comparison of simple potential functions for simulating liquid water. *J. Chem. Phys.* 79:926–935.

20. Lindorff-Larsen, K., S. Piana, …, D. E. Shaw. 2010. Improved side-chain torsion potentials for the Amber ff99SB protein force field. *Proteins.* 78:1950–1958.

21. Jämbeck, J. P., and A. P. Lyubartsev. 2012. Derivation and systematic validation of a refined all-atom force field for phosphatidylcholine lipids. *J. Phys. Chem. B.* 116:3164–3179.

22. Horn, A. H. 2014. A consistent force field parameter set for zwitterionic amino acid residues. *J. Mol. Model.* 20:2478.

23. Hess, B. 2008. P-lincs: a parallel linear constraint solver for molecular simulation. *J. Chem. Theory Comput.* 4:116–122.

24. Essman, U., L. Perera, …, L. G. Pedersen. 1995. A smooth particle mesh Ewald method. *J. Chem. Phys.* 103:8577–8593.

25. Darden, T., D. York, and L. Pedersen. 1993. Particle mesh Ewald - an N.log(N) method for Ewald sums in large systems. *J. Chem. Phys.* 98:10089–10092.

26. Berendsen, H. J. C., J. P. M. Postma, …, J. R. Haak. 1984. Molecular dynamics with coupling to an external bath. *J. Chem. Phys.* 81:3684–3690.

27. Nose, S. 1984. A molecular dynamics method for simulations in the canonical ensemble. *Mol. Phys.* 52:255–268.

28. Parinello, M., and A. Rahman. 1981. Polymorphic transitions in single crystals - a new molecular dynamics method. *J. Appl. Phys.* 52:7182–7190.

29. Dobrev, P., S. Donnini, …, H. Grubmüller. 2017. Accurate three states model for amino acids with two chemically coupled titrating sites in explicit solvent atomistic constant pH simulations and pK(a) calculations. *J. Chem. Theory Comput.* 13:147–160.

30. Tribello, G. A., M. Bonomi, …, G. Bussi. 2014. PLUMED 2: new feathers for an old bird. *Comput. Phys. Commun.* 185:604–613.

31. Grossfield, A. 2003. WHAM: the weighted histogram analysis method. http://membrane.urmc.rochester.edu/wordpress/?page_id=126.

32. Aldeghi, M., J. P. Bluck, and P. C. Biggin. 2018. Absolute alchemical free energy calculations for ligand binding: a beginner's guide. *In* Computational Drug Discovery and Design. M. Gore and U. B. Jagtap, eds. Springer New York, pp. 199–232.

33. Aldeghi, M., A. Heifetz, …, P. C. Biggin. 2017. Predictions of ligand selectivity from absolute binding free energy calculations. *J. Am. Chem. Soc.* 139:946–957.

34. Boresch, S., F. Tettinger, …, M. Karplus. 2003. Absolute binding free energies: a quantitative approach for their calculation. *J. Phys. Chem. B.* 107:9535–9551.

35. Klimovich, P. V., M. R. Shirts, and D. L. Mobley. 2015. Guidelines for the analysis of free energy calculations. *J. Comput. Aided Mol. Des.* 29:397–411.

36. Seeliger, D., and B. L. de Groot. 2010. Protein thermostability calculations using alchemical free energy simulations. *Biophys. J.* 98:2309–2316.

37. Gapsys, V., S. Michielssens, …, B. L. de Groot. 2015. pmx: automated protein structure and topology generation for alchemical perturbations. *J. Comput. Chem.* 36:348–354.

38. Rocklin, G. J., D. L. Mobley, …, P. H. Hünenberger. 2013. Calculating the binding free energies of charged species based on explicit-solvent simulations employing lattice-sum methods: an accurate correction scheme for electrostatic finite-size effects. *J. Chem. Phys.* 139:184103.

39. Gur, M., E. Zomot, …, I. Bahar. 2015. Energy landscape of LeuT from molecular simulations. *J. Chem. Phys.* 143:243134.

40. Shaikh, S. A., J. Li, …, E. Tajkhorshid. 2013. Visualizing functional motions of membrane transporters with molecular dynamics simulations. *Biochemistry.* 52:569–587.

41. Razavi, A. M., G. Khelashvili, and H. Weinstein. 2018. How structural elements evolving from bacterial to human SLC6 transporters enabled new functional properties. *BMC Biol.* 16:31.

42. Immadisetty, K., J. Hettige, and M. Moradi. 2017. What can and cannot be learned from molecular dynamics simulations of bacterial proton-coupled oligopeptide transporter GkPOT? *J. Phys. Chem. B.* 121:3644–3656.

43. Latorraca, N. R., N. M. Fastman, …, L. Feng. 2017. Mechanism of substrate translocation in an alternating access transporter. *Cell.* 169:96–107.e12.

44. Galochkina, T., M. Ng Fuk Chong, …, C. Etchebest. 2019. New insights into GluT1 mechanics during glucose transfer. *Sci. Rep.* 9:998.

45. Selvam, B., S. Mittal, and D. Shukla. 2018. Free energy landscape of the complete transport cycle in a key bacterial transporter. *ACS Cent. Sci.* 4:1146–1154.

46. Wambo, T. O., L. Y. Chen, …, G. Perry. 2017. Affinity and path of binding xylopyranose unto E. coli xylose permease. *Biochem. Biophys. Res. Commun.* 494:202–206.

47. Terry, D. S., R. A. Kolster, …, S. C. Blanchard. 2018. A partially-open inward-facing intermediate conformation of LeuT is associated with $Na^+$ release and substrate transport. *Nat. Commun.* 9:230.

48. Kyrychenko, A., N. M. Lim, …, A. S. Ladokhin. 2018. Refining protein penetration into the lipid bilayer using fluorescence quenching and molecular dynamics simulations: the case of diphtheria toxin translocation domain. *J. Membr. Biol.* 251:379–391.

49. Matsunaga, Y., T. Yamane, …, A. Kidera. 2018. Energetics and conformational pathways of functional rotation in the multidrug transporter AcrB. *eLife.* 7:e31715.

50. Lazaridis, T., and G. Hummer. 2017. Classical molecular dynamics with mobile protons. *J. Chem. Inf. Model.* 57:2833–2845.

51. Khelashvili, G., N. Stanley, …, H. Weinstein. 2015. Spontaneous inward opening of the dopamine transporter is triggered by PIP2-regulated dynamics of the N-terminus. *ACS Chem. Neurosci.* 6:1825–1837.

52. Koldsø, H., H. E. Autzen, …, B. Schiøtt. 2013. Ligand induced conformational changes of the human serotonin transporter revealed by molecular dynamics simulations. *PLoS One.* 8:e63635.

53. Wisedchaisri, G., M. S. Park, …, T. Gonen. 2014. Proton-coupled sugar transport in the prototypical major facilitator superfamily protein XylE. *Nat. Commun.* 5:4521.

54. Zhao, C., and S. Y. Noskov. 2011. The role of local hydration and hydrogen-bonding dynamics in ion and solute release from ion-coupled secondary transporters. *Biochemistry.* 50:1848–1856.

55. Shaikh, S. A., and E. Tajkhorshid. 2010. Modeling and dynamics of the inward-facing state of a Na+/Cl- dependent neurotransmitter transporter homologue. *PLoS Comput. Biol.* 6:e1000905.

56. Huang, Y., W. Chen, …, J. Shen. 2016. Mechanism of pH-dependent activation of the sodium-proton antiporter NhaA. *Nat. Commun.* 7:12940.

57. Yue, Z., W. Chen, …, J. Shen. 2017. Constant pH molecular dynamics reveals how proton release drives the conformational transition of a transmembrane efflux pump. *J. Chem. Theory Comput.* 13:6405–6414.

**Biophysical Journal, Volume *117***

## Supplemental Information

# Proton Control of Transitions in an Amino Acid Transporter

**Zhiyi Wu, Irfan Alibay, Simon Newstead, and Philip C. Biggin**

A

B

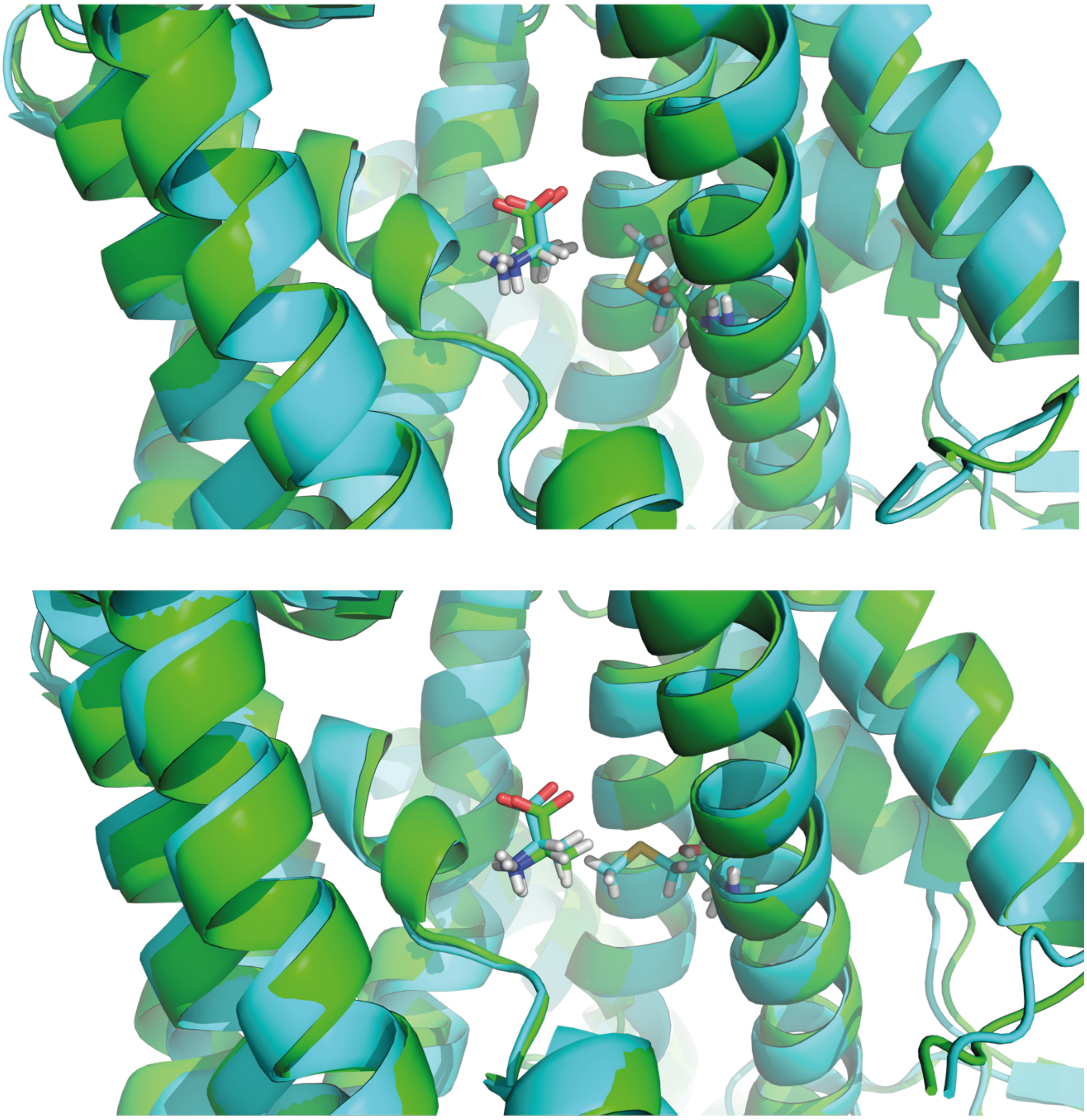

**Figure S1**. Comparison of M321S mutant structures to wildtype. (**A**) M321S mutant structure (green) overlaid onto wildtype (blue) inward-occluded structure (RMSD = 1.27 Å). (**B**) Comparison of M321S mutant (green) to wildtype (blue) for the inward-open conformation (RMSD = 1.10 Å).

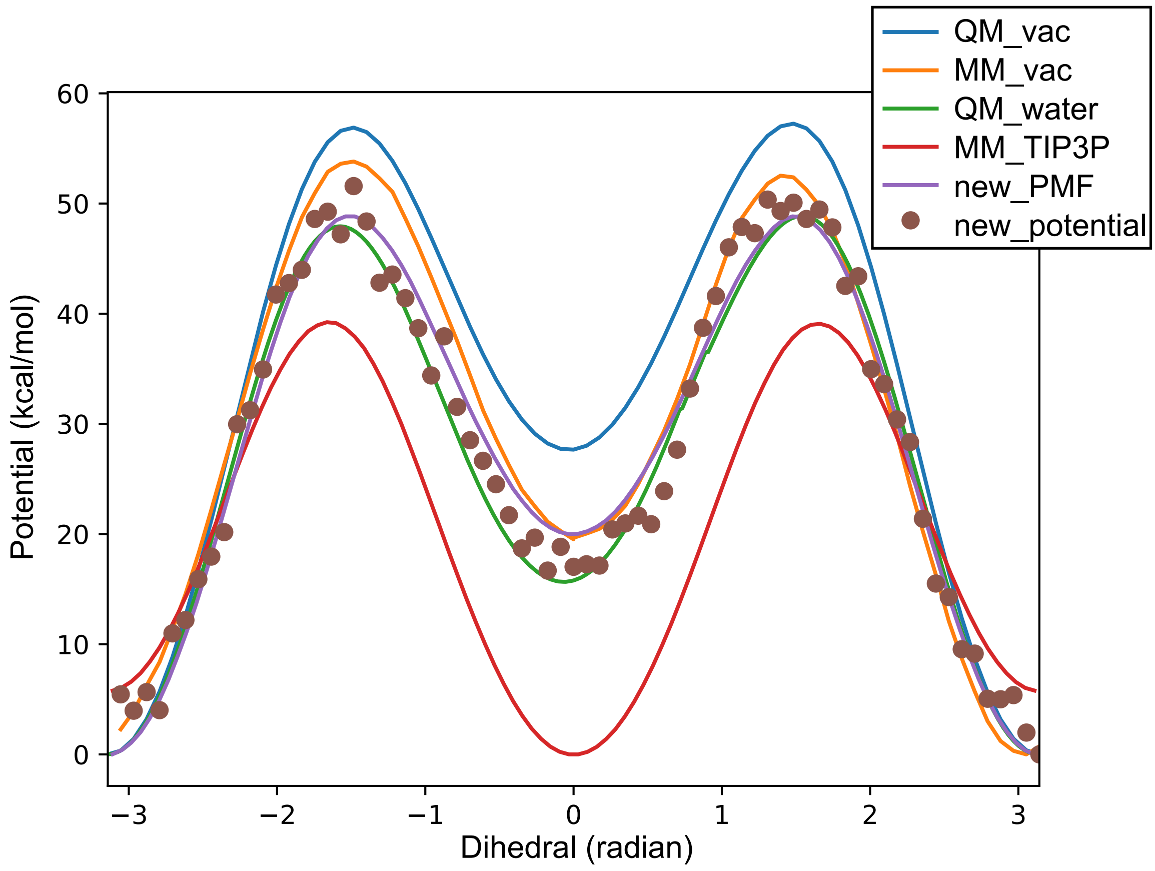


**Figure S2**. Potential energy surface of the C-C-O-H dihedral for a protonated glutamate residue (atoms CG-CD-OE2-HE2). Though the potential energy surface of the C-C-O-H dihedral in the Amber99SB-ILDN force field (orange line) is relatively consistent with the QM energy surface calculated at the HF/6-31G(d) level (blue line) in vacuum, the minimum dihedral angle is flipped by 180 degrees when solvated with TIP3P explicit water (red line), which is different from the QM energy surface calculated at the same level of theory with implicit PCM water (green line). The refitted dihedral reproduces the QM energy surface both in terms of the total system potential (brown dot) or the PMF profile (purple line) when solvated with TIP3P water. The new dihedral potential is represented using the Ryckaert-Bellemans dihedral, which has the functional form

$$\frac{1}{2}[F_1(1+\cos\varphi)+F_2(1-\cos 2\varphi)+F_3(1+\cos 3\varphi)]$$

where $F_1$ = 34.8164509, $F_2$ = -13.0191847 and $F_3$ = -21.7972662 (kJ $mol^{-1}$).

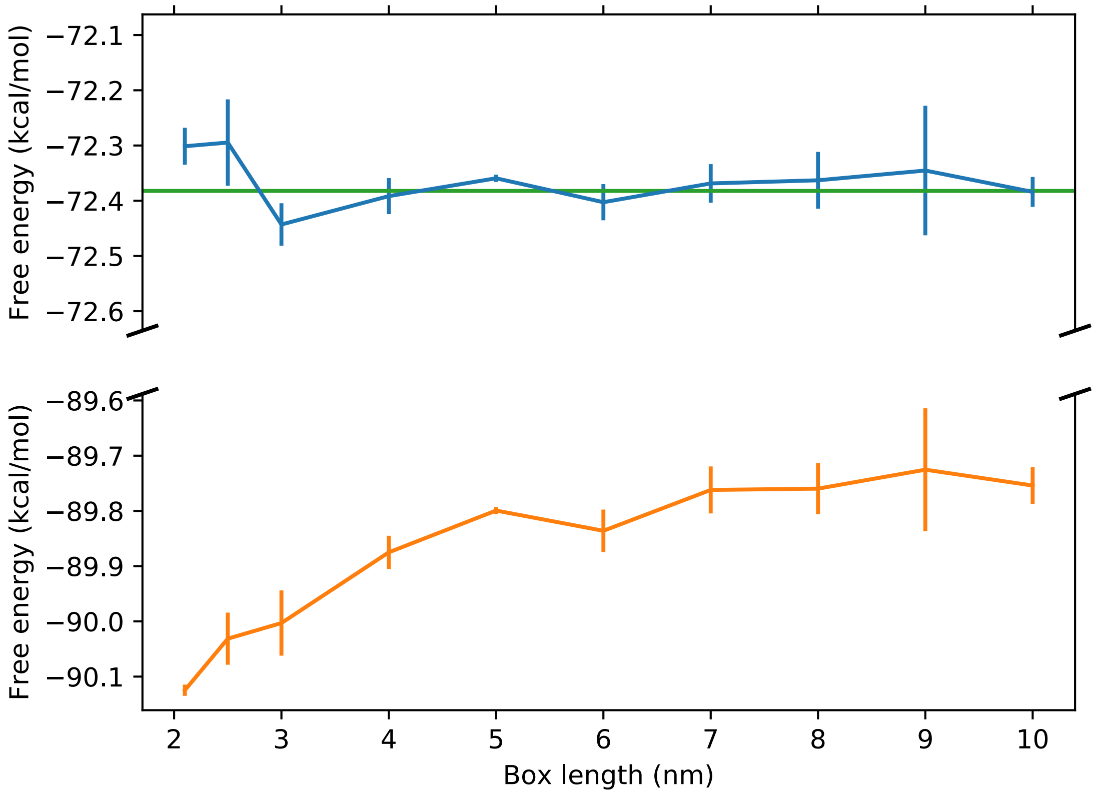


**Figure S3.** Correction of the finite size error for the chloride ion charge annihilation free energy. The charge annihilation free energy exhibits a significant size dependent trend (orange line), which can be corrected for (blue line) such that there is no longer any size dependence. The final chloride ion charge annihilation free energy is taken as the average of the corrected free energy from box length 3 nm to 10 nm (green line).

A

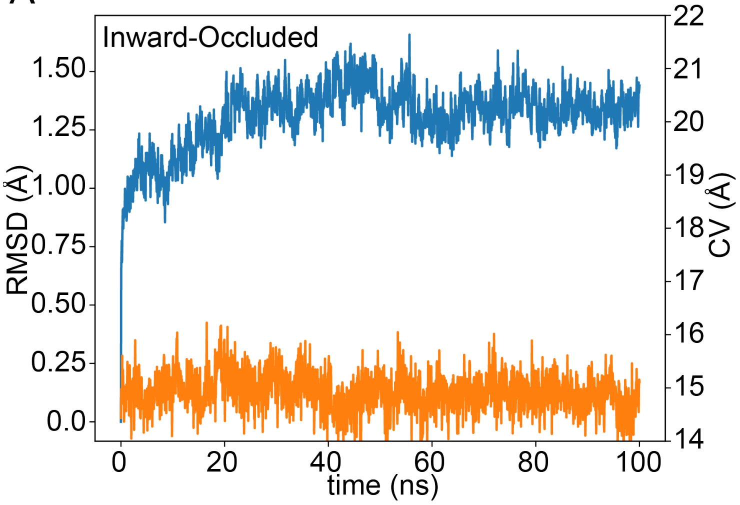


B

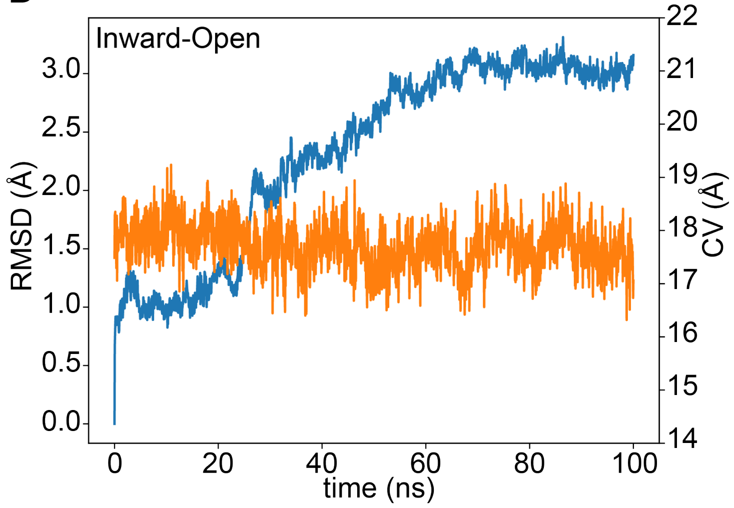


**Figure S4**. Dynamic behaviour of the (A) unbiased simulation of the inward-occluded protonated protein in complex with alanine and (B) unbiased simulation of the inward-open deprotonated protein in complex with alanine. The root mean squared deviation (RMSD) of the Cα atoms with respect to the first simulation frame (blue line) as well as the collective variable (orange line) defined as the distance between the center of mass of residues 113 to 117 and the center of mass of residues 235 to 239.

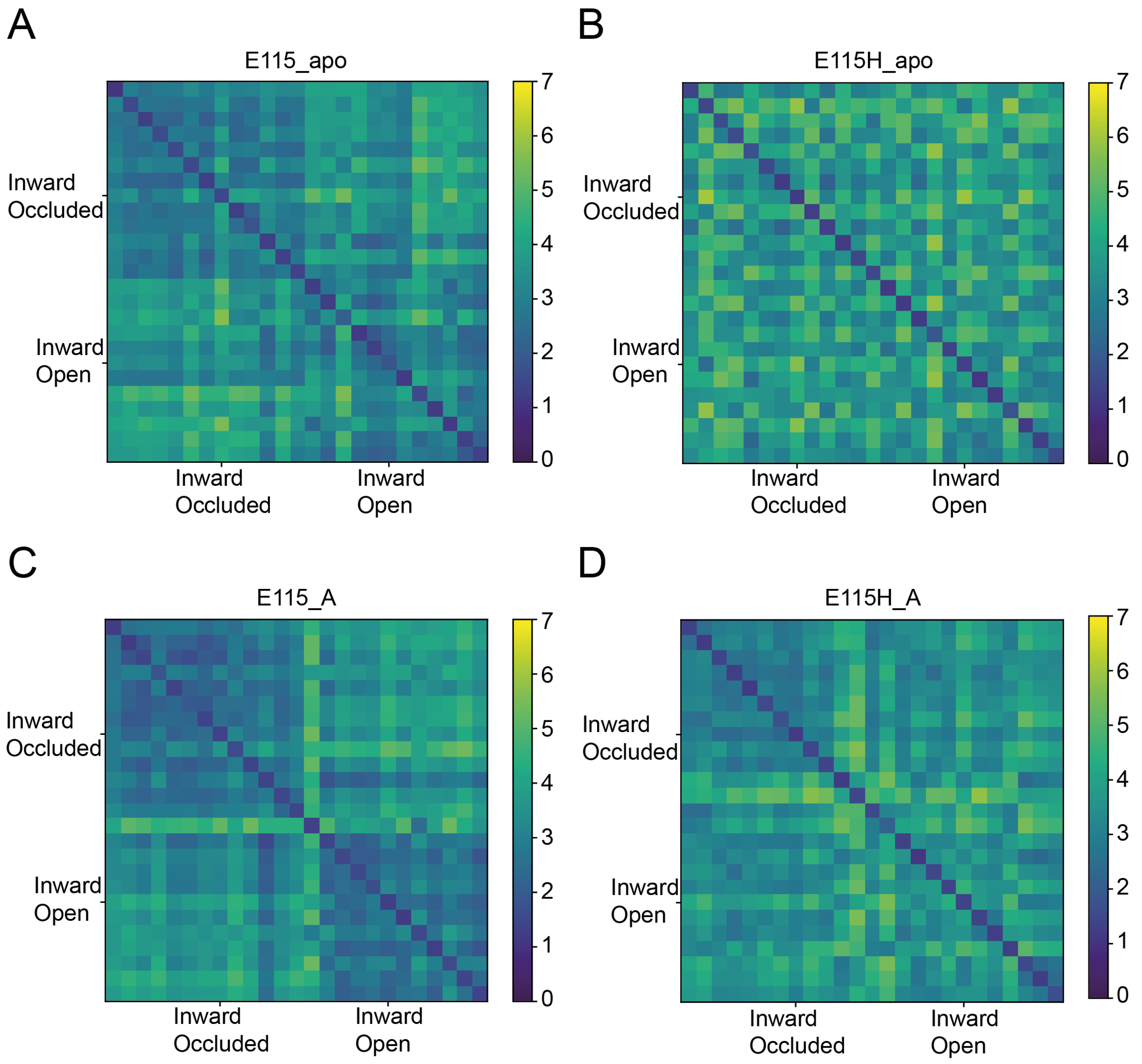


**Figure S5.** Average Cα RMSD of each CV window with respect to the end frame of all CV windows. Each pixel (*i*,*j*) is the average RMSD of trajectory in window *i* with respect to the last frame of window *j*.

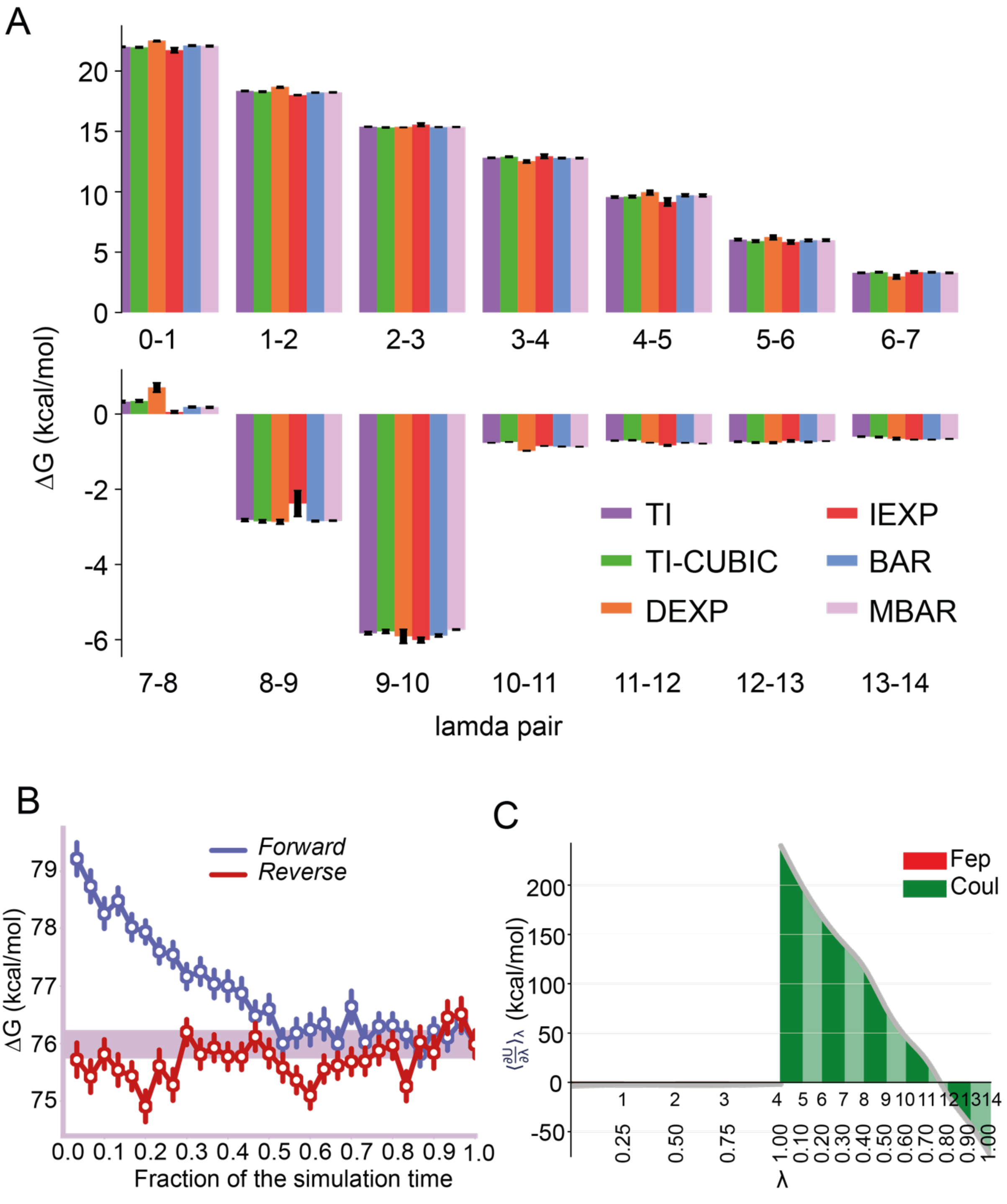


**Figure S6**. Convergence assessment measure for protonation of E115 in the apo inward occluded state. (**A**) Bar chart showing the free energy difference between adjacent lambda states evaluated via several methods along with the estimated error. (**B**). Free energy convergence in the sampling time axis. The free energy difference is shown in both forward series, where the value is the result of proportion of the data used (i.e. 0~10%, 0~20%) and in reverse fashion where the result is the reverse

proportion of the data (i.e. 90~100%, 80~100%). The simulation is stopped when the forward and reverse series converged to within 1 kcal/mol. (**C**). The gradient of the free energy with respect to lambda estimated for each lambda windows. The silver line is the interpolation of the gradient via the cubic spline and the area under the curve is the free energy integrated along the lambda axis. Coul indicates the free energy difference of alchemically changing the charge of the residue from protonated glutamate to deprotonated glutamate, while fep indicates the free energy change of the bonded, angle and dihedral terms as well as the annihilation of the vdw of the extra proton.

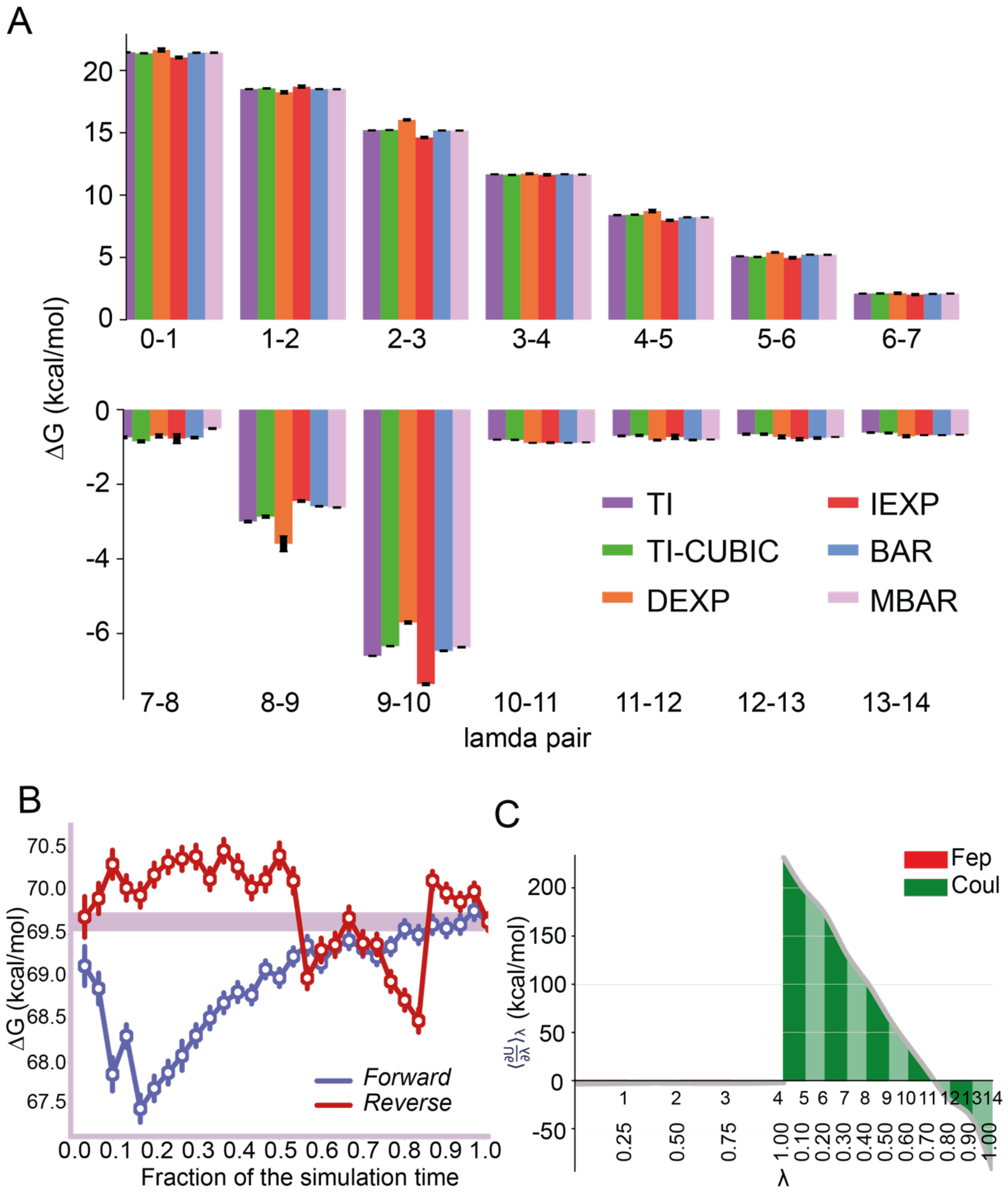


**Figure S7**. Convergence assessment measure for protonation of E115 in the apo inward open state. Panels **A-C** are as for Figure S5.

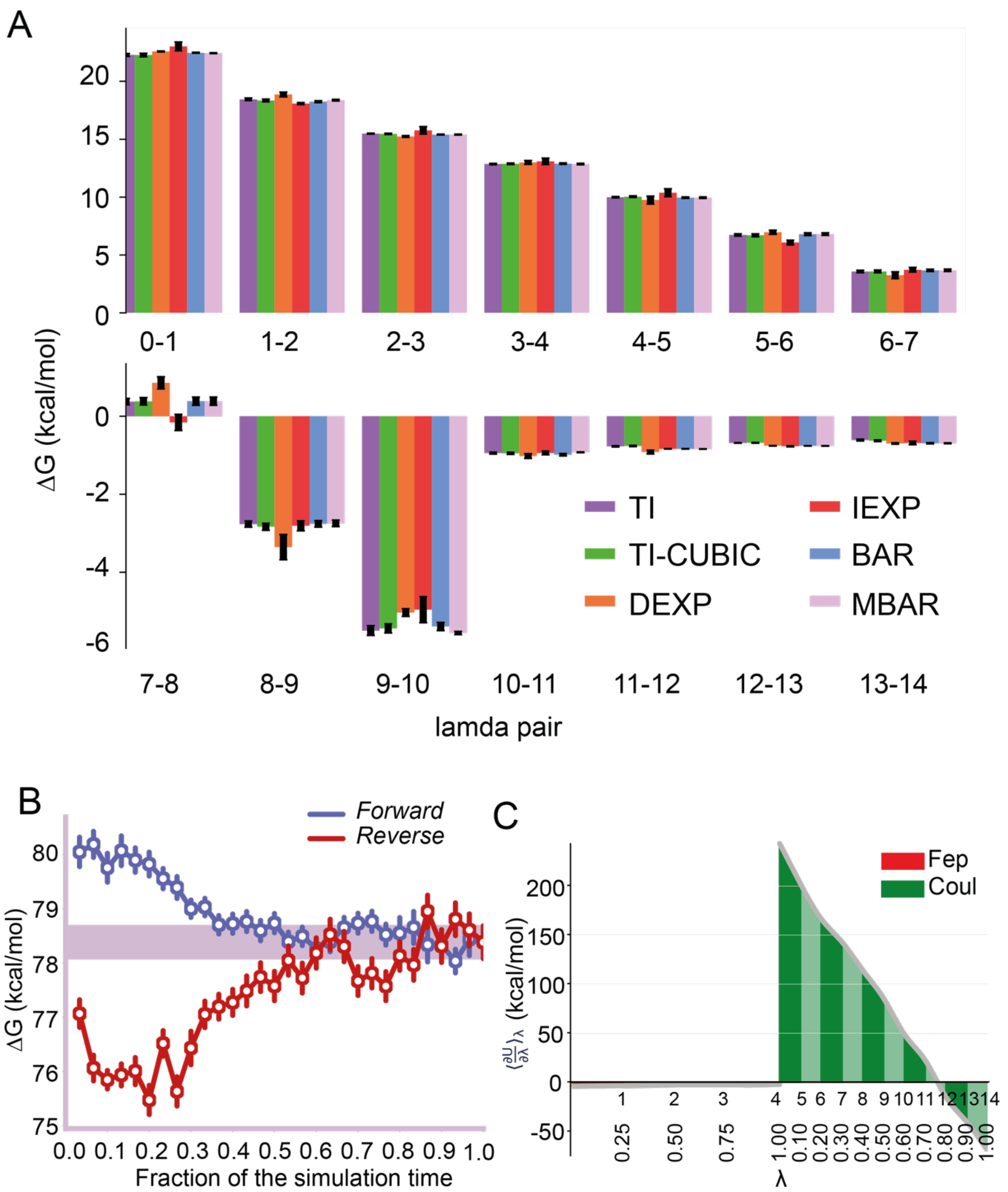


**Figure S8**. Convergence assessment measure for protonation of E115 in the alanine bound inward occluded state. Panels **A-C** are as for Figure S5.

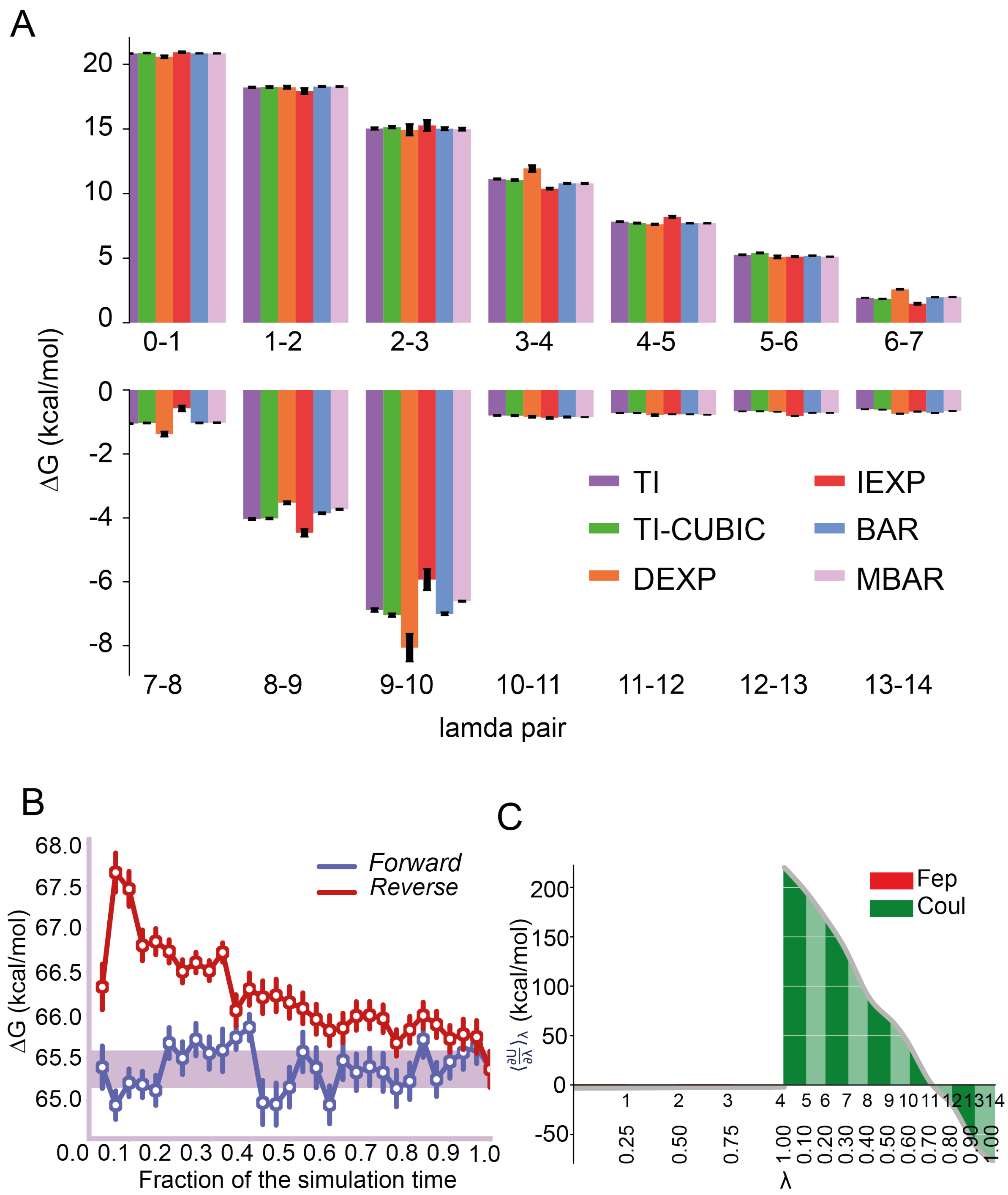


**Figure S9**. Convergence assessment measure for protonation of E115 in the alanine bound inward open state. Panels **A-C** are as for Figure S5.

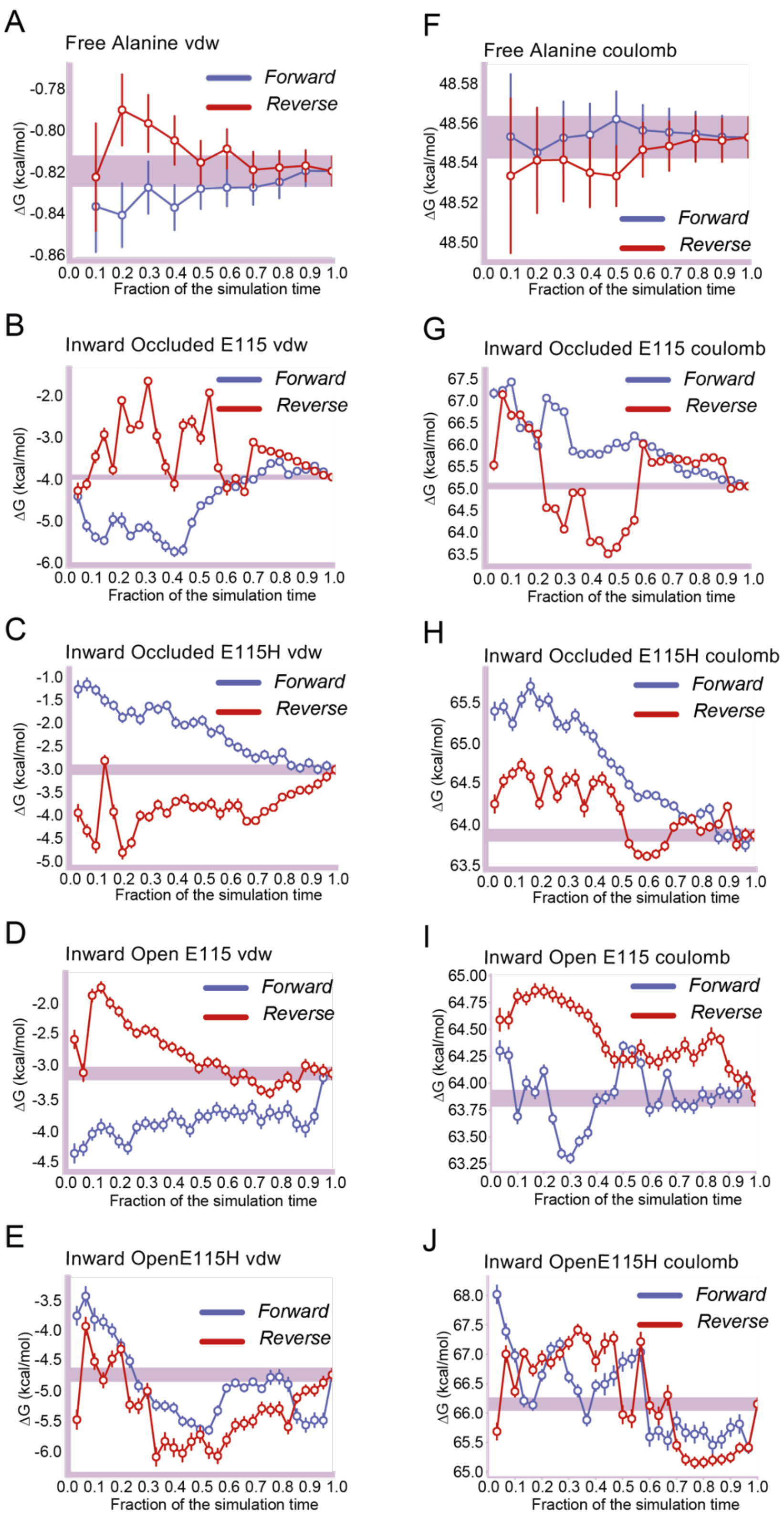


**Figure S10**. ΔG estimates as a fraction of simulation time. Panels **A-E** and **F-J** are vdw and coulombic contributions respectively. **A**, free alanine in solution; **B**, inward occluded E115; **C**, inward occluded E115H; **D**, inward open E115; **E**, inward open E115H; **F**, free alanine in solution; **G**, inward occluded E115; **H**, inward occluded E115H; **I**, inward open E115; and **J**, inward open E115H.
